# Discovering the Potentials of Four Phage Endolysins to Combat Gram-Negative Infections

**DOI:** 10.3389/fmicb.2021.748718

**Published:** 2021-10-13

**Authors:** Daria V. Vasina, Nataliia P. Antonova, Igor V. Grigoriev, Victoria S. Yakimakha, Anastasiya M. Lendel, Maria A. Nikiforova, Andrei A. Pochtovyi, Timofey A. Remizov, Evgeny V. Usachev, Natalia V. Shevlyagina, Vladimir G. Zhukhovitsky, Mikhail V. Fursov, Vasiliy D. Potapov, Aleksei M. Vorobev, Andrey V. Aleshkin, Aleksei I. Laishevtsev, Valentine V. Makarov, Sergey M. Yudin, Artem P. Tkachuk, Vladimir A. Gushchin

**Affiliations:** ^1^Laboratory of Pathogen Population Variability Mechanisms, N. F. Gamaleya National Research Center for Epidemiology and Microbiology, Ministry of Health of the Russian Federation, Moscow, Russia; ^2^Translational Biomedicine Laboratory, N. F. Gamaleya National Research Center for Epidemiology and Microbiology, Ministry of Health of the Russian Federation, Moscow, Russia; ^3^Faculty of Biology, Lomonosov Moscow State University, Moscow, Russia; ^4^Laboratory of Indication and Ultrastructural Analysis of Microorganisms, N. F. Gamaleya National Research Center for Epidemiology and Microbiology, Ministry of Health of the Russian Federation, Moscow, Russia; ^5^Russian Medical Academy of Continuing Professional Education (RMANPO), Ministry of Public Health, Moscow, Russia; ^6^Aerobiological Laboratory, State Research Center for Applied Microbiology and Biotechnology, Obolensk, Russia; ^7^Laboratory of Clinical Microbiology and Biotechnology of Bacteriophages, G. N. Gabrichevsky Moscow Research Institute for Epidemiology and Microbiology, Moscow, Russia; ^8^Laboratory for Diagnostics and Control of Antibiotic Resistance of the Most Clinically Significant Pathogens of Animals, Federal State Budget Scientific Institution “Federal Scientific Centre VIEV” (FSC VIEV), Moscow, Russia; ^9^Center for Strategic Planning of the Ministry of Health of the Russian Federation, Moscow, Russia

**Keywords:** endolysin, Gram-negative bacteria, antimicrobial agents, local infection, animal models, safety

## Abstract

Endolysin-based therapeutics are promising antibacterial agents and can successfully supplement the existing antibacterial drugs array. It is specifically important in the case of Gram-negative pathogens, e.g., ESKAPE group bacteria, which includes *Enterococcus faecium*, *Staphylococcus aureus*, *Klebsiella pneumoniae*, *Acinetobacter baumannii*, *Pseudomonas aeruginosa*, and *Enterobacter* species, and are highly inclined to gain multiple antibiotic resistance. Despite numerous works devoted to the screening of new lytic enzymes and investigations of their biochemical properties, there are significant breaches in some aspects of their operating characteristics, including safety issues of endolysin use. Here, we provide a comprehensive study of the antimicrobial efficacy aspects of four Gram-negative bacteria-targeting endolysins LysAm24, LysAp22, LysECD7, and LysSi3, their *in vitro* and *in vivo* activity, and their biological safety. These endolysins possess a wide spectrum of action, are active against planktonic bacteria and bacterial biofilms, and are effective in wound and burn skin infection animal models. In terms of safety, these enzymes do not contribute to the development of short-term resistance, are not cytotoxic, and do not significantly affect the normal intestinal microflora *in vivo*. Our results provide a confident base for the development of effective and safe candidate dosage forms for the treatment of local and systemic infections caused by Gram-negative bacterial species.

## Introduction

The rapid increase of the discovery of new antibiotics during the 1950–60s is now replaced with moderate development of these therapeutics because of the growing commercial risks associated with the emergence of bacterial resistance. This has resulted in a rising focus on the development of derivatives of known antibiotics rather than discovery of antimicrobials with fundamentally new mechanisms of action. However, a significant drawback of such an approach is concluded in its inability to withstand the increase of bacterial antibiotic resistance. Thus, an active search for nonconventional approaches to limit or eliminate the emergence of resistant infectious agents’ burden is carried out (Theuretzbacher et al., 2020).

During the last decades, the pharmaceutical industry set sights on biotherapeutics and biotechnologically derived antimicrobials, primarily proteins, peptides, mABs, and their artificial derivatives (Theuretzbacher and Piddock, 2019). Among new treatment strategies is the use of peptides and enzymes, specifically lysins and endolysins, with bacterial cell wall disruptive activity from the outside (Grabowski et al., 2021). On the one hand, this resembles a classical and effective mechanism of action of several classes of antibiotic agents (glycopeptide antibiotics, β-lactams, and polymyxins); on the other hand, it is believed that the enzybiotic mechanism of action oriented toward specific conservative targets will lead to the less dramatic and slow emergence of bacterial resistance (Grishin et al., 2020).

Dozens of peptidoglycan-degrading molecules acting against different bacterial species, either Gram-positive or Gram-negative, are reported annually. However, very few of them reach the advanced preclinical and clinical phases of development, among which are recombinant lysins against *Staphylococcus aureus* SAL200 (tonabacase; Jun et al., 2017) and *CF*-301 (exebacase; Fowler et al., 2020) for systemic administration and topical endolysins for the treatment of atopic dermatitis infected with *S. aureus* (Gladskin; Totté et al., 2017) and for prevention of nasal MRSA colonization (GangaGen, ClinicalTrials.gov Identifier: NCT01746654). Endolysin-based formulations against Gram-positive bacteria (especially *S. aureus*) predominate in late preclinical or clinical experience despite the fact that recent studies have also shown effectiveness of endolysins against highly resistant Gram-negative bacteria-caused infections (Gutiérrez and Briers, 2021).

Such a gap is the result of specific hurdles accompanying the development of Gram-negative acting enzymes, among which are (i) troubles with the transition from *in vitro* antibacterial activity results to *in vivo* level. The antibacterial action *in vitro* is usually evaluated under controlled cell growth conditions or growth stage, and the experiments do not fully reflect the real or even model conditions (Oliveira et al., 2018). (ii) The potential for bacterial resistance to lysins is still under question, although it was not shown for any of endolysins previously using the serial passage experiments (Grishin et al., 2020). (iii) Safety profiles *in vitro* and *in vivo* as well as pharmacokinetics of endolysins are not yet well understood. The sizes and origin of proteins restrict their distribution in the body; however, their ability to affect cells and blood components has not been evaluated. (iv) The immunogenicity of reusable lysin-based preparations is also not clarified yet. The protein origin of lysins should induce immune response in mammals causing reduced activity of preparations with each subsequent application. Thus, the development of neutralizing antibodies is of concern for repeated use in humans (Murray et al., 2021). The success of further developments of lysin-based preparations is based on reliable data on their efficiency and safety, solving the raised questions.

Previously, we characterized the *in vitro* activity of *Myoviridae* bacteriophage endolysins LysAm24, LysECD7, and LysSi3 (Antonova et al., 2019), representing diverse domain organizations (single-domain vs. two-domain) and different predicted mechanisms of action (lysozyme vs. peptidase activities). Likewise, LysAp22 lysozyme-like endolysin was obtained and tested. All of the assayed molecules were capable of lysing Gram-negative clinical isolates – representatives of the ESKAPE pathogen group. About 5–50μg/ml of individual endolysins was enough to eradicate growing cells over more than five orders of magnitude. Importantly, recombinant enzymes revealed bactericidal activity without any specialized OM penetration approach or additives, suggesting their potential in the development of medicines with reliable effectiveness. Here, we assess the potentials and risks of the application of these four Gram-negative bacteria-targeting endolysins with different structures and mechanisms of action in *in vitro* and *in vivo* experiments. We report the results of *in vitro* and *in vivo* efficacy studies against bacterial strains and biofilms, including skin and burn wound animal models. Also, we review the safety aspects of endolysin use, assessing the impact on intestinal microbiome, interaction with immune response, and the ability of bacteria to acquire resistance toward the enzymes.

## Materials and Methods

### Bacterial Strains

The bacterial strains used in the study included laboratory strains and clinical isolates of Gram-negative representatives of the ESKAPE group of pathogens from the collection of the N.F. Gamaleya Federal Research Center for Epidemiology and Microbiology, Ministry of Health of the Russian Federation, from the State Collection of Pathogenic Microorganisms and Cell Cultures “SCPM-Obolensk” and the Collection of Gabrichevsky Moscow Research Institute of Epidemiology and Microbiology (Supplementary Table S1). All the strains were stored at −80°C and cultivated in the appropriate medium at 37°C, at 250rpm overnight before performing the assays.

### Ethics Statements

All animal experiments were conducted following the relevant guidelines for the care and use of laboratory animals. The Ethics Committee of the State Research Center for Applied Microbiology and Biotechnology (Obolensk, Russia) approved the *in vivo* studies (Veterinary Protocol No. VP-2019/9 of SRCAMB Bioethics Committee). The outbred female mice were used for the *in vivo* assessment of endolysins’ impact on the intestinal microbiome. The outbred female mice and male Wistar rats were used in effectiveness experiments for skin wound and burn wound models correspondingly. Two female Californian rabbits (4.5–5.0kg) were used for the immunization. Animals were purchased from Andreevka Nursery (Russia). All animals were housed in separate cages with controlled temperature (20–24°C) and humidity (45–65%) and fed with a balanced diet and water *ad libitum*.

### Recombinant Expression and Purification of Proteins

Recombinant endolysins LysAm24 (NCBI AN: APD20282.1), LysAp22 (NCBI AN: CCH57765.1), LysECD7 (NCBI AN: ASJ80195.1), and LysSi3 (NCBI AN: ARK07361.1) were obtained as described previously (Antonova et al., 2019). The initial coding sequences were artificially synthesized in a pAL-TA commercial vector (Evrogen, Moscow, Russia). Thereafter, endolysins’ ORFs were amplified from pAL-TA clones and integrated into the expression vector pET-42b(+; Evrogen, Moscow, Russia), resulting in four pET42b-endolysin-8his plasmids. All constructs were checked for errors *via* Sanger sequencing. The expression vectors were introduced into the competent *Escherichia coli* cells, strain BL21(DE3) pLysS (chloramphenicol resistance), using a heat shock transformation protocol.

The *E. coli* exponential cultures were induced with 1mM β-D-1-thiogalactopyranoside at 37°C for 3h, centrifuged, and disrupted by sonication. The cells were harvested by centrifugation (6,000×*g* for 10min at 4°C) and resuspended in lysis buffer (20mM Tris–HCl, 250mM NaCl, and 0.1mM EDTA, pH=8.0), incubated with 100μg/ml lysozyme at room temperature for 30min, and disrupted by sonication. The cell debris was removed by centrifugation (10,000×*g* for 30min at 4°C), and the supernatant was filtered through a 0.2-μm filter. The proteins were purified on an NGC Discovery™ 10 FPLC system (Bio-Rad, Hercules, CA, United States) with HisTrap FF column (GE Healthcare, Munich, Germany) pre-charged with Ni^2+^ ions. The filtered lysate was mixed with 30mM imidazole and 1mM MgCl_2_ and loaded on the column pre-equilibrated with binding buffer (20mM Tris–HCl, 250mM NaCl, and 30mM imidazole, pH=8.0). The fractions were eluted using a linear gradient to 100% elution buffer (20mM Tris–HCl, 250mM NaCl, and 500mM imidazole pH 8.0). The protein fractions were dialyzed against 20mM Tris–HCl (pH=7.5).

The purity of the proteins was determined by 16% SDS-PAGE, and protein concentrations were measured using a spectrophotometer (Implen NanoPhotometer, Implen, München, Germany) at 280nm and calculated using predicted extinction coefficients [0.840, 0.831, 1.4595, and 1.0289 (mg/ml)^−1^ cm^−1^ for LysAm24, LysAp22, LysECD7, and LysSi3, respectively]. All proteins were lyophilized and stored at −80°C.

### Endolysin Activity Assay

Overnight bacterial cultures were diluted in a Mueller–Hinton broth and grown to the exponential phase (OD_600_=0.6). Subsequently, the cells were harvested by centrifugation (3,000×*g*, 10min, RT) and resuspended in 20mM Tris–HCl (pH=7.5) buffer to a final density of approximately 10^5^–10^6^ cells/ml. Afterward, 100μl of the bacterial suspension and 100μl of the protein at the final 100-μg/ml concentration were mixed in 96-well plate wells, with a buffer without endolysins used as the negative control. The mixtures were incubated at 37°C for 5, 10, 30, 60, or 120min with shaking at 200rpm, diluted 10-fold in PBS (pH=7.4), and plated onto Mueller–Hinton agar. The number of surviving bacterial colonies was counted after overnight incubation at 37°C. All experiments were performed in triplicate, and the antibacterial activity was expressed as follows: bactericidal activity (%)=100% – (CFUexp/CFUcont)×100%, where CFU_exp_ is the number of bacterial colonies in the experimental culture plates and CFU_cont_ is the number of bacterial colonies in the control culture plates. Antibacterial activity on the definite strain was regarded as meaningful when it was higher than 33%.

The spectrum of activity was studied as described above with a 30-min incubation period at the 100-μg/ml final protein concentration. Antibiotic susceptibility analysis of some bacterial strains was performed by the broth microdilution method using Mueller–Hinton broth, according to ISO recommendations (ISO 20776-1; Clinical laboratory testing and *in vitro* diagnostic test systems – Susceptibility testing of infectious agents and evaluation of the performance of antimicrobial susceptibility testing devices – part 1. Geneva, Switzerland: International Organization for Standardization, 2006). Results were interpreted according to The European Committee on Antimicrobial Susceptibility Testing (breakpoint tables for interpretation of MICs and zone diameters, Version 11.0, 2021).

### Scanning Electron Microscopy of Planktonic Cells

An overnight *Acinetobacter baumannii* strain Ts 50-16 bacterial culture was diluted in LB broth, grown to an exponential phase (OD_600_=0.6), and harvested by centrifugation. Subsequently, the cell pellet was washed twice with distilled water and resuspended in water to a final density of approximately 10^8^cells/ml (McFarland 0.5). Afterward, 150μl of the bacterial suspension was mixed with 150μl of the protein diluted in water to the 50-μg/ml concentration and incubated at 37°C for 30min with shaking at 200rpm. Buffer without endolysins was used as the negative control. Mixtures were fixed by adding 600μl of 10% formalin for 24h. Next, 10μl of mixtures was placed on a slide and dried at RT conditions. The samples were mounted to stubs and sputter-coated with a gold layer (5nm) in an SPI-Module Sputter/Carbon Coater System (SPI Inc., West Chester, PA, United States). The sputtering samples were analyzed using a scanning Quanta 200 3D Dual Beam Electron Microscope (FEI Company, Hillsboro, OR, United States) using the high vacuum mode (10kV).

### Microscopy of Biofilms

Sterile glass coverslips (Hampton Research, Aliso Viejo, CA, United States) were plunged into overnight *A. baumannii* Ts 50-16 culture in tryptic soy broth (TSB) medium in Petri dishes and incubated at 37°C for 24h without shaking for biofilm formation. Then, slides were washed with distilled water three times and air-dried. Three slides were left intact, three were submerged into 20mM Tris–HCl (pH=7.5) control buffer, other slides were submerged into 100μg/ml LysAm24, LysAp22, LysECD7, or LysSi3 solutions, with three slides for each solution, respectively, for 2h at RT. Afterward, all slides were again washed with distilled water three times and air-dried. One slide from each group was fixed in 10% formalin vapors for 24h and analyzed using scanning electron microscopy after coating with a gold layer, as described above. Another slide from each group was imprinted on LB agar to assess bacterial viability. The remaining slides were stained with 0.1% aqueous solution of crystal violet for 20min at RT, washed three times with water, and dried. These slides were analyzed using an Axiostar Plus Transmitted Light Microscope (Zeiss AG, Jena, Germany) at ×400 magnification.

### Biofilm Reduction Assay

Endolysin antibiofilm activity was assessed on mature biofilms of *A. baumannii* Ts 50-16, *Klebsiella pneumoniae* Ts 104-14, and *Pseudomonas aeruginosa* Ts 38-16, as described before (Fursov et al., 2020) with modifications. Overnight bacterial cultures in TSB medium were diluted 1:50 in fresh TSB, with 100μl added to wells of 96-well sterile polystyrene cell culture plates, and incubated for 24h at 37°C, 250rpm, to allow biofilm formation. After incubation, the wells’ contents with planktonic cells were shaken off, and the plate was washed with 200μl of PBS (pH=7.4) three times and air-dried for approximately 10min. Then, 100μl of endolysin solutions in the concentration of 100 and 1mg/ml or 20mM Tris–HCl buffer (pH=7.5) as negative control was added to the wells and incubated at 37°C, 250rpm, for 2h. After incubation, wells were twice washed with 200μl of PBS (pH=7.4) and air-dried. Washed biofilms were stained with 0.1% aqueous solution of crystal violet for 20min at RT followed by triple rinsing with PBS. The remaining stain was re-solubilized in 200μl of 33% acetic acid, and OD_600_ of the obtained solution was measured using SPECTROstar NANO (BMG LABTECH, Ortenberg, Germany). All experiments were performed in triplicate. Values were normalized by dividing by OD_600_ of untreated biofilm.

The interpretation of the level of biofilm formation was done according to Stepanović et al. (2007). Weak biofilm was defined at ODc<ODbf ≤2×ODc, moderate biofilm at 2×ODc<ODbf ≤4×ODc, and strong biofilm at 4×ODc<ODbf, where ODc is the cutoff value calculated as three SDs above the mean OD of the negative control and ODbf=the optical density of bacterial biofilm stained with crystal violet.

### Determination of Bacterial Resistance

Resistance development was tested using repeated exposure of endolysins on bacterial cultures in plate lysis assay and antibacterial assay on initial and passed strains of *A. baumannii* Ts 50-16 and *K. pneumoniae* Ts 104-14.

Overnight bacterial cultures were diluted in LB broth and grown at 37°C, 250rpm, to an exponential phase (OD_600_=0.6). Subsequently, 200μl of the bacterial suspension was spread over a Petri dish with LB agar and air-dried at RT. Then, 10μl of 1mg/ml endolysin solutions or control 20mM Tris–HCl buffer (pH=7.5) were dropped onto the bacterial lawn, and the dish was treated for 16–18h at 37°C. The next day, bacteria from the sublethal lysis zone with a not fully cleared lawn were scraped and incubated in LB broth to exponential phase at 37°C with constant shaking to generate a new bacterial lawn for the next passage. There were 20 passages of *A. baumannii* and *K. pneumoniae* cultures during the experiment. Afterward, cultures from initial and passed strains were used to assess the antibacterial activity of endolysins as described before in 50μg/ml concentration. All experiments were performed in triplicate.

### Cytotoxicity Assay

Cytotoxicity was measured for HEK293 (ATCC-CRL-1573) cells by MTT assay. Cells were seeded at 4.5×10^4^ cells/well in 96-well plates and cultivated in 100μl of DMEM medium supplemented with 10% fetal bovine serum, 2mM glutamine, 50U/ml penicillin, and 50μg/ml streptomycin for 24h in a humidified atmosphere of 5% CO_2_ and 95% air at 37°C. Afterward, the medium was removed and 50μl of endolysin serial dilutions in DMEM (from 2,000 to 31.1μg/ml) was added into each well. DMEM was used as a negative control, and 0.1% Triton X-100 was used as a positive control. Mixtures were incubated for 1h (37°C, 5% CO_2_), and the cells were then stained with 10% MTT solution in DMEM (final stain concentration is 0.5mg/ml) for 4h (37°C, 5% CO_2_). After incubation, the content of the wells was replaced with 100μl of DMSO and the absorbance of the solutions at the wavelength of 570nm was measured using SPECTROstar NANO (BMG LABTECH). All experiments were performed in triplicate, and the viability of the HEK293 cells was estimated as follows: Viability (%)=(OD_exp_−OD_min_)/(OD_max_−OD_min_)×100%, where OD_exp_ is OD_570_ in the experimental culture wells, OD_min_ is OD_570_ in the positive control (0.1% Triton X-100) culture wells, and OD_max_ is OD_570_ in the negative control (DMEM) culture wells.

### Hemolytic Assay

The hemolytic activity of endolysins was determined against human red blood cells (RBC). Fresh human blood was obtained in a heparin-containing tube and was centrifuged at 500*g* for 10min, 4°C. The pellet was washed three times with PBS buffer, and a solution of 10% RBC in PBS was prepared. An RBC solution was mixed in a ratio of 1:1 with endolysin solutions in concentrations of 100 and 1mg/ml in 20mM Tris–HCl buffer (pH=7.5). PBS buffer was used as a negative control, and 0.1% Triton X-100 was used as a positive control. The reaction mixtures were incubated for 1h at 37°C and harvested by centrifugation (500*g*, 10min, 4°C), and the absorbance at the wavelength of 405nm of the supernatants was measured. All experiments were performed in triplicate, and the hemolysis rate was estimated as follows: Hemolysis (%)=(OD_exp_−OD_PBS_)/(OD_Triton_−OD_PBS_)×100%, where OD_exp_ is OD_405_ in the experimental mixtures, OD_PBS_ is OD_405_ in the negative control (PBS) mixtures, and OD_Triton_ is OD_405_ in the positive control (0.1% Triton X-100) mixtures.

### Neutralizing Antibody Effect Assessment

The neutralization effect of specific anti-LysAm24, LysAp22, LysECD7, and LysSi3 antibodies on enzymes antibacterial activity was tested in the presence of hyperimmune serum or purified antibodies.

To obtain hyperimmune serum, rabbits were immunized with 0.25mg of each endolysin eight times with 10-day interval resulting in a 3-month immunization period. For the first immunization, the solutions of each antigen were emulsified with equal volumes of Freund’s complete adjuvant (Sigma, St. Louis, MO, United States) and injected subcutaneously to 8–9 sites on the back, and re-immunizations were repeated with Freund’s incomplete adjuvant (Sigma, United States). Rabbit sera were sampled 10days after the last injection, and the titers of specific antibodies (>100,000) were determined using the ELISA method. Serum from rabbits without immunization was used as a control.

The neutralization effect of immunized serum was estimated by mixing 20μl of 1mg/ml of endolysin solutions with 80μl of immunized or control rabbit serum, followed by incubation at 37°C for 10min. Afterward, 100μl of exponential *A. baumannii* Ts 50-16 culture in 20mM Tris–HCl buffer (pH=7.5) with a cell density of 10^5^–10^6^CFU/ml was added to each 96-well plate well, with a buffer without endolysins as the negative control. Next, the mixtures were incubated at 37°C for 30min with shaking at 200rpm, diluted 10-fold in PBS (pH=7.4), and plated onto an LB agar. The number of surviving bacterial colonies was counted after overnight incubation at 37°C. All of the experiments were performed in triplicate, and the antibacterial activity was expressed as described before.

The neutralization effect of specific antibodies was estimated after antibody purification from hyperimmune serum with affinity chromatography. Each endolysin in 0.05 М MES (pH=5.5) and 0.15 М NaCl buffer was conjugated to aminoethyl-sepharose sorbent by overnight incubation with 200μg/ml EDC [1-ethyl-3-(3-dimethylaminopropyl)carbodiimide] at RT with rotation at 15rpm and equilibrated with PBS, 0.5 М NaCl, and 0.1% NaN_3_. Then, hyperimmune rabbit serum was supplied with 0.5 М NaCl, centrifuged (30min, 10,000×*g*), loaded on the column, and washed with the same buffer. The specific antibody fractions were eluted using 0.1 М CH_3_COOH and 0.5 М NaCl (рН=2.5–2.8) buffer, and the pH was immediately adjusted to neutral by adding 1M Tris–HCl pH 8.0. Afterward, the buffer was exchanged to PBS (pH=7.4) using a PD-10 gel-filtration column (GE Healthcare, Chicago, IL, United States), and the solution was concentrated with Amicon® Ultra-15 Centrifugal Filter Units, 30-kDa MWCO (Millipore, Bedford, MA, United States). The antibody concentration was measured using a spectrophotometer (Implen NanoPhotometer, Implen, Germany) at 280nm while antibody-specific activity was confirmed by indirect ELISA.

Endolysin solutions (50μl) in 20mM Tris–HCl buffer (pH=7.5) were mixed with 50μl of antibodies in PBS in mass ratios of 1:1, 1:2, and 1:5 (the PBS solution was used as a positive control) and incubated at 37°C for 10min without agitation in a 96-well plate. Endolysin concentrations in wells were 25μg/ml for LysAm24 and LysAp22, 50μg/ml for LysECD7, and 100μg/ml for LysSi3. Thereafter, 100μl of exponential *A. baumannii* Ts 50-16 culture in 20mM Tris–HCl buffer (pH=7.5) was added to each 96-well plate well, with a 20mM Tris–HCl buffer (pH=7.5) without endolysins as the negative control. Further, the experiment was performed as described above.

### Assessment of Impact of Endolysins on Intestinal Microbiome

#### *In vitro* Tests

Endolysins’ effect on microbiota representatives was tested *in vitro* on eight strains of *Bifidobacterium* sp. and three strains of *Lactobacillus* sp. (Supplementary Table S1). These bacteria are representatives of normal microbial flora and are a form of probiotic drugs intended for correction of the microflora of three age groups of patients with dysbiosis of the gastrointestinal tract: children under the age of 3years (patent RU 2491331), those from 3 to 14years (patent RU 2491335), and people over 14years old (patent RU 2491336).

Freeze-dried bacterial cultures were rehydrated in 1ml specific for *Bifidobacterium* or *Lactobacillus* media (State Research Center for Applied Microbiology and Biotechnology, Russia), and their 10-fold dilutions in fresh liquid medium were cultivated at 37°C for 24h in anaerobic conditions. Grown bacterial colonies were reinoculated in fresh medium twice and then plated onto agar in an anaerobic culture apparatus. Next, bacterial colonies were resuspended in normal saline to OD_600_=0.6 and diluted in 20mM Tris–HCl (pH=7.5) buffer to a final density of approximately 10^5^–10^6^ cells/ml. Endolysin solutions (1ml) with a protein concentration of 1mg/ml were mixed in glass tubes with bacterial suspension (9ml), incubated for 30min at 37°C, 10-fold diluted in a liquid medium, and incubated for 48h at 37°C in anaerobic conditions. The final proteins concentration was 100μg/ml. Afterward, the number of surviving bacterial colonies in tubes was counted. All of the experiments were performed in triplicate. The experimental workflow is shown in Supplementary Figure S1.

#### *In vivo* Evaluation

Endolysins’ effect on bacterial intestinal microbiome was also assessed *in vivo* using a murine model of outbred female mice (22–27g). Before the experiment, animals were randomized into experimental groups (*n*=5 per group), which were intraperitoneally injected with 0.5ml of endolysin in the concentration of 1mg/ml (500μg/mouse) every 24h for 5days and one control group (*n*=8) that received 0.5ml of 20mM Tris–HCl (pH=7.5) the same way. On the seventh day of the experiment (2days after the last injection of LysAm24, LysECD7, LysAp22, and LysSi3), mice were sacrificed by CO_2_ inhalation and the parietal microbiota was investigated using the 16S sequencing of samples containing intestine with excrements. All samples were stored at −80°C until the analysis.

#### 16S rRNA Gene Sequencing of Parietal Microbiome

Frozen intestine samples from each of the animals were homogenized with TissueLyser II (Qiagen, Manchester, United Kingdom) in PBS buffer, and DNA isolation was performed using the DNeasy PowerSoil Kit (Qiagen, United Kingdom) according to the manufacturer’s instructions. DNA quality was assessed using agarose gel, and the yield was measured with a Qubit 3 Fluorometer (Invitrogen, Carlsbad, CA, United States).

The hypervariable V3-V4 region of the bacterial 16S rRNA gene was amplified using the forward primer NR_16S_341F (5'-TCGTCGGCAGCGTCAGATGTGTATAAGAGACAGTGCCTACGGGN BGCASCAG-3') and reverse primer NR_16S_806R (5'-GTCTCGTGGGCTCGGAGATGTGTATAAGAGACAGCCGGACTACNV GGGTWTCTAATCC-3'). Primers were modified with forward overhang 5'-TCGTCGGCAGCGTCAGATGTGTATAAGAGACAG-3' and with reverse overhang 5'-GTCTCGTGGGCTCGGAGATGTGTATAAGAGACAG-3' (Nextera tag sequences) for dual index library preparation. Amplicons were pooled in equal concentrations and purified using magnetic bead extraction to remove unwanted products. The purified libraries were quantified with 2100 Bioanalyzer (Agilent, Santa Clara, CA, United States), diluted as recommended by Illumina MiSeq library preparation guide, and sequenced with a MiSeq Reagent Kit v3 (2×300cycles).

#### Sequencing Data Analysis

Raw Illumina fastq files were demultiplexed and quality filtered (quality score not less Q30), chimeric reads were removed, and paired amplicon sequencing reads were joined using the QIIME pipeline (version 1.9.1). Operational taxonomic units (OTUs) were identified from the trimmed sequences at 97% identity level as implemented in QIIME with the Silva reference database (version 132). The OTUs to family and genus were then collapsed, and the relative abundance of each bacterium in every sample was calculated. The following statistical analyses were based on family- and genus-level data. The fold changes in microbial species after the endolysins administration in comparison to Tris-buffer injections samples were calculated for genus–taxa level.

### Animal Efficacy Studies

#### Skin Wound Model

Skin infection was induced with a *K. pneumoniae* Ts 141-14 clinical isolate. The backs of outbred female mice (20–22*g*) were shaved and infected subcutaneously with 100μl of 2×10^8^CFU/ml bacterial suspension in PBS buffer. During the first day after the infection, the abscess was formed and ruptured spontaneously, resulting in an open wound in the place of injection. At 24h post-infection (PI), the treatment had begun. The infected area was either treated epicutaneously with 100μl of 20mM Tris–HCl (pH=7.5) control buffer (*n*=10) or treated with 100μl of each endolysin solution in the concentration of 500μg/mouse (*n*=10 mice in each group), left untreated (*n*=20). Mice were treated every 24h for 5days.

To access the endolysins’ therapeutic effect, the dynamics of wound healing and microbial contamination were measured after 1, 3, and 7days PI. CFU counts of *K. pneumoniae* were estimated using the wound swabs with sterile cotton balls wetted with saline. Inoculations were serially diluted and plated on LB agar. After 7days of the experiment, the mice were sacrificed by CO_2_ inhalation, and the infected dermal grafts of the wound area and animal spleens were excised, weighted, and homogenized in PBS. The solutions were serially diluted and plated on LB agar, and bacterial CFU loads were assessed after overnight incubation at 37°C.

#### Burn Wound Model

For the burn wound model, the *P. aeruginosa* Ts 38-16 clinical isolate was used. The backs of 8–10-week-old male Wistar rats (180–210*g*) were shaved with an electric razor. To induce burn injury, a copper plate with a surface area 150mm^2^ was heated to 300°C and then applied on the shaved backs for 30s. To restore the water–electrolyte balance, 1ml of PBS buffer was injected subcutaneously to the burned areas. Five minutes later, the burn was infected with 3.5×10^10^CFU/ml of *P. aeruginosa* (100μl in PBS). After 24h PI, the infected burns were either left untreated (*n*=20), treated with 100μl of 20mM Tris–HCl (pH=7.5) control buffer (*n*=10), or treated with 100μl of endolysin solution in the concentration of 2,500μg/rat (*n*=10 in each group). The rats were treated epicutaneously every 24h for 5days.

To access the endolysins’ therapeutic effect, the dynamics of wound size and microbial contamination were observed after 1, 3, and 7days PI. CFU counts of *P. aeruginosa* were estimated using the wound swabs as mentioned above using the selective Pseudomonas Agar B Medium (for fluorescein; HiMedia, Mumbai, India). After 7days of experiment, the rats were sacrificed by CO_2_ inhalation. Infected dermal grafts of the burn area and animal spleens were excised, weighted, and homogenized in PBS. The solutions were serially diluted and plated on Agar B Medium, and bacterial CFU were counted after overnight incubation at 37°C.

### Statistical Analysis

The data were analyzed using GraphPad Prism 7.0 software. A value of *p*<0.05 was considered statistically significant. The methods used for comparison tests are indicated in figure and table captions.

## Results

### *In vitro* Antimicrobial Activity of Endolysins

**Figure 1:**
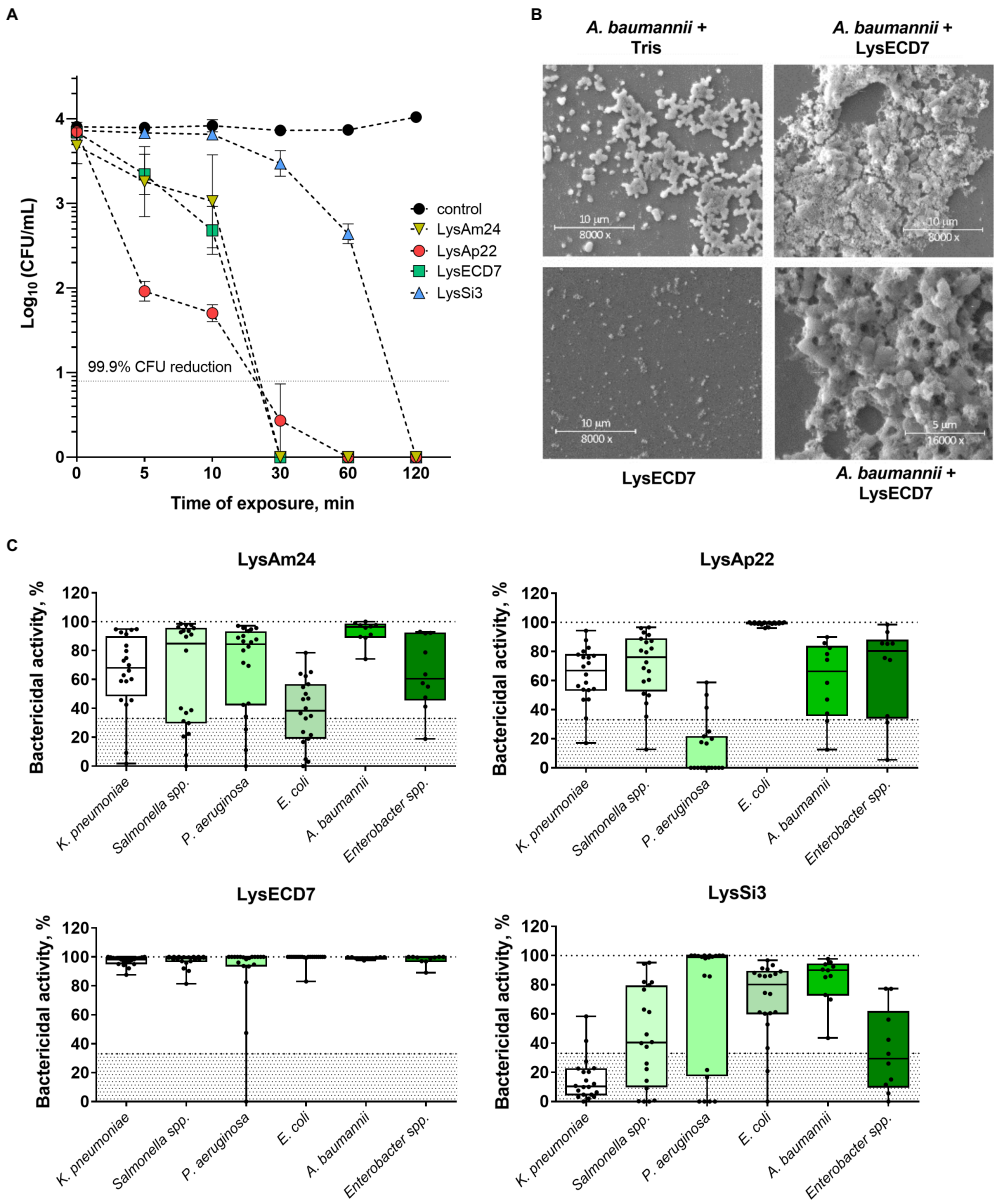
*In vitro* efficacy of four endolysins. **(A)** Time-kill curve of endolysins activity in 100μg/ml concentration against logarithmic-phase growing planktonic *Acinetobacter baumannii* Ts 50-16 cells. Data are shown as mean±SEM. **(B)** Scanning electron microscopy of *A. baumannii* Ts 50-16 planktonic cells exposed to 50μg/ml of LysECD7 or Tris–HCl buffer for 30min, or protein in Tris–HCl buffer. **(C)** LysAm24, LysECD7, LysAp22, and LysSi3 (100μg/ml) spectra of activity after a 30-min incubation with logarithmic-phase growing bacteria. Each dot represents a single strain within the species, lines, and medians; boxes, IQR whiskers, and min–max. The 33% activity cutoff is indicated with a dotted line.

The enzymes’ bactericidal activity was evaluated using the microplate assay measuring CFU counts after incubation of *A. baumannii* cell suspension with endolysins. All lysins exhibited rapid activity, capable of eliminating 100% of cells within 1h except for LysSi3, which required 2h for the total elimination of bacteria (Figure 1A). It was suggested to compare time required to kill 99.9% of *A. baumannii* planktonic cells by endolysins in equal concentrations and, in fact, characterize its bactericidal activity (Pfaller et al., 2004; Balouiri et al., 2016). For LysAp22, LysECD7, LysAm24, and LysSi3 in concentration 100μg/ml, it was 22.7, 23.3, 24.1, and 99.6min, respectively. Cell lysis of the exponentially growing bacteria after the incubation with lysins was confirmed with scanning electron microscopy (Figure 1B). The formation of debris accompanying osmosis-mediated cell lysis resulted in сell disruption of bacteria due to the loss of peptidoglycan integrity under lysin action.

The spectrum of activity of LysAm24, LysAp22, LysECD7, and LysSi3 was estimated against 100 clinical isolates and strains of Gram-negative pathogens, including multidrug-resistant *K. pneumoniae*, *Salmonella* sp., *P. aeruginosa*, *Escherichia coli*, *A. baumannii*, and *Enterobacter* sp. (Figure 1C; Supplementary Table S1). For all endolysins, we observed similar results that indicated a broad range of sensitive bacterial species, with LysECD7 being the most active among enzymes with the highest rate of antimicrobial activity (99% of strains with activity above 33%). LysAm24 was active against 81% of strains, LysAp22 – 66%, LysSi3 – 54%. A concentration of 100μg/ml was enough to eradicate growing bacteria up to five orders of magnitude. Among the investigated species, *A. baumannii* was the most sensitive to lysins.

**Figure 2:**
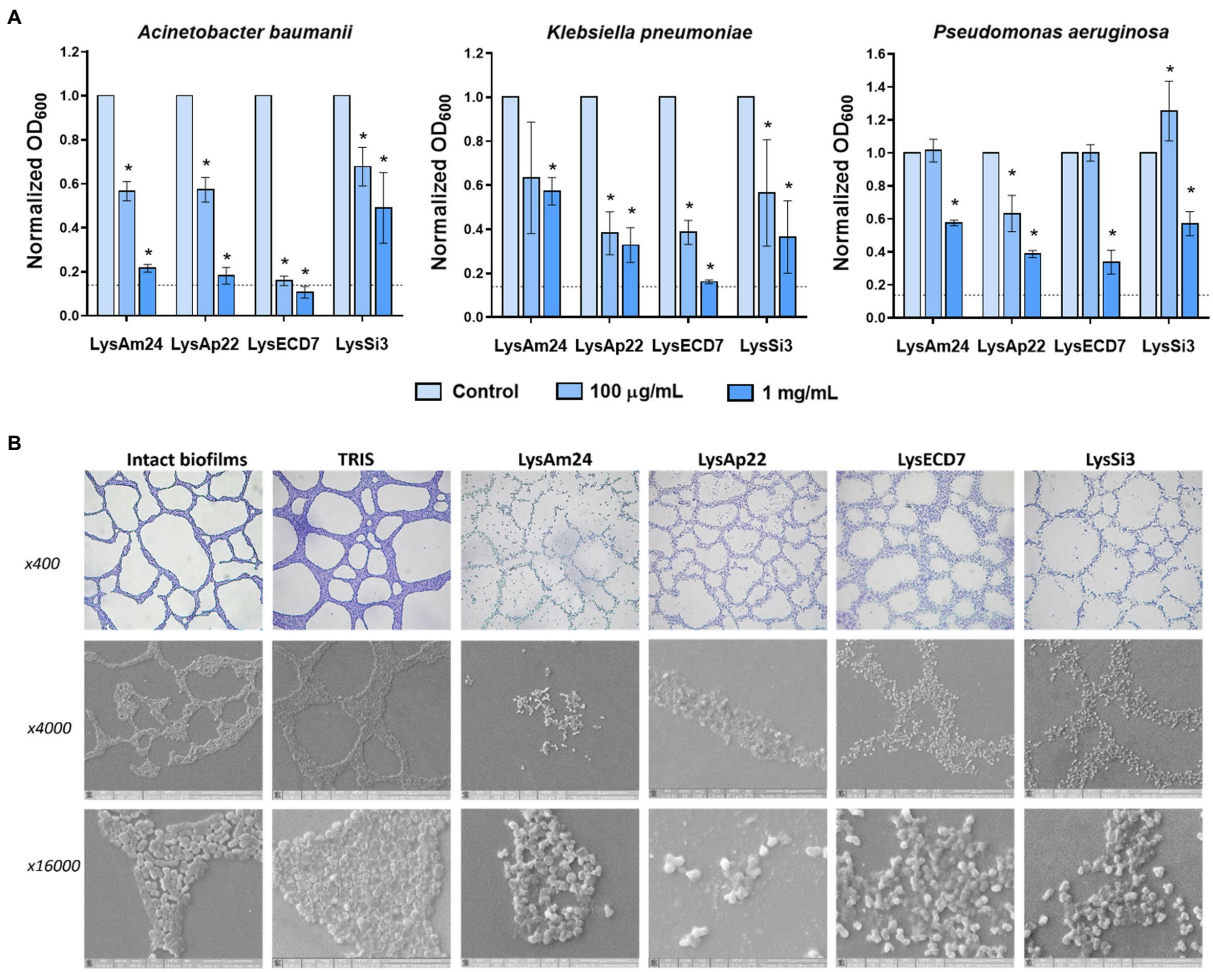
Disruption of bacterial biofilms by endolysins. **(A)** Antibiofilm activity of endolysins against *Klebsiella pneumoniae* Ts 104-14, *A. baumannii* Ts 50-16, and *Pseudomonas aeruginosa* Ts 38-16 biofilms stained with crystal violet. OD_600_ was measured after 2h of exposure to endolysins. Dash line indicates the cutoff value calculated as three SDs above the mean OD of the negative control. Data are shown as mean±SD. Asterisk (^*^) indicates significant difference in optical density compared to control without the enzyme treatment of biofilm (*p*<0.05, one-way ANOVA). **(B)** Light and scanning electron microscopy images of lysis of *A. baumannii* Ts 50-16 preformed biofilms on glass slides after a 2-h exposure to endolysins in 100mg/ml concentration.

The bacterial biofilms’ disruptive activity was also investigated. The ability of endolysins to eliminate 24-h preformed strong biofilms of *A. baumannii* Ts 50-16, *K. pneumoniae* Ts 104-14, and *P. aeruginosa* Ts 38-16 in microtiter plates is shown in Figure 2A.

Two hours of exposure to 100 and 1mg/ml of endolysins resulted in a dose-dependent decrease of optical density of biofilms. LysECD7 again had the most pronounced effect compared to LysAm24, LysAp22, and LysSi3 and was capable of eliminating biofilms. Also, it was noticed that *A. baumannii* biofilms were exposed to endolysin activity most significantly, which is consistent with data on planktonic cells. To directly observe the interactions of antimicrobial enzymes with bacterial films, a combination of light and scanning electron microscopy was applied (Figure 2B). The breakdown of the structural integrity of the bacterial film occurs under the action of lysins, while the film matrix is destroyed and only individual shapeless cells are indicated. Nondisrupted cells were also observed, and their viability was confirmed by glass slide imprinting on a solid medium after treatment with the endolysin solution. Growth of single CFUs was found after exposure to LysECD7, LysAp22, and LysSi3, and no bacterial growth in the case of LysAm24.

### *In vitro* Safety of Recombinant Endolysins

#### Bacterial Resistance Development Toward Endolysins

The ability of bacteria to develop resistance after repeated exposure to sublethal endolysin concentrations was studied on *A. baumannii* Ts 50-16 and *K. pneumoniae* Ts 104-14 clinical isolates in a plate lysis assay. Antibacterial activity of lysins (50μg/ml) against initial and passed strains after 20 passages did not differ in the case of *K. pneumoniae* Ts 104-14 or *A. baumannii* Ts 50-16. Thus, no resistance appearance to Gram-negative endolysins with different enzymatic activities accompanying the loss of antibacterial effect was estimated.

#### Cytotoxicity and Hemolysis

Safety of LysAm24, LysAp22, LysECD7, and LysSi3 endolysins was assessed in cytotoxicity and hemolytic assays. The impact on eukaryotic HEK293 cells in the concentration range from 2,000 to 31μg/ml was not significant, and cell viability was approximately 100% in almost all proteins. Significant cell viability reduction was reported in the 2,000-μg/ml concentration of LysAm24 and LysSi3; it was 58.4 and 70.9%, respectively (Supplementary Figure S2). The percent of hemolysis of human blood cells did not exceed 0.5% in the 1-mg/ml concentration, which is very insufficient (Supplementary Figure S3).

#### Interaction With Specific IgG

**Figure 3:**
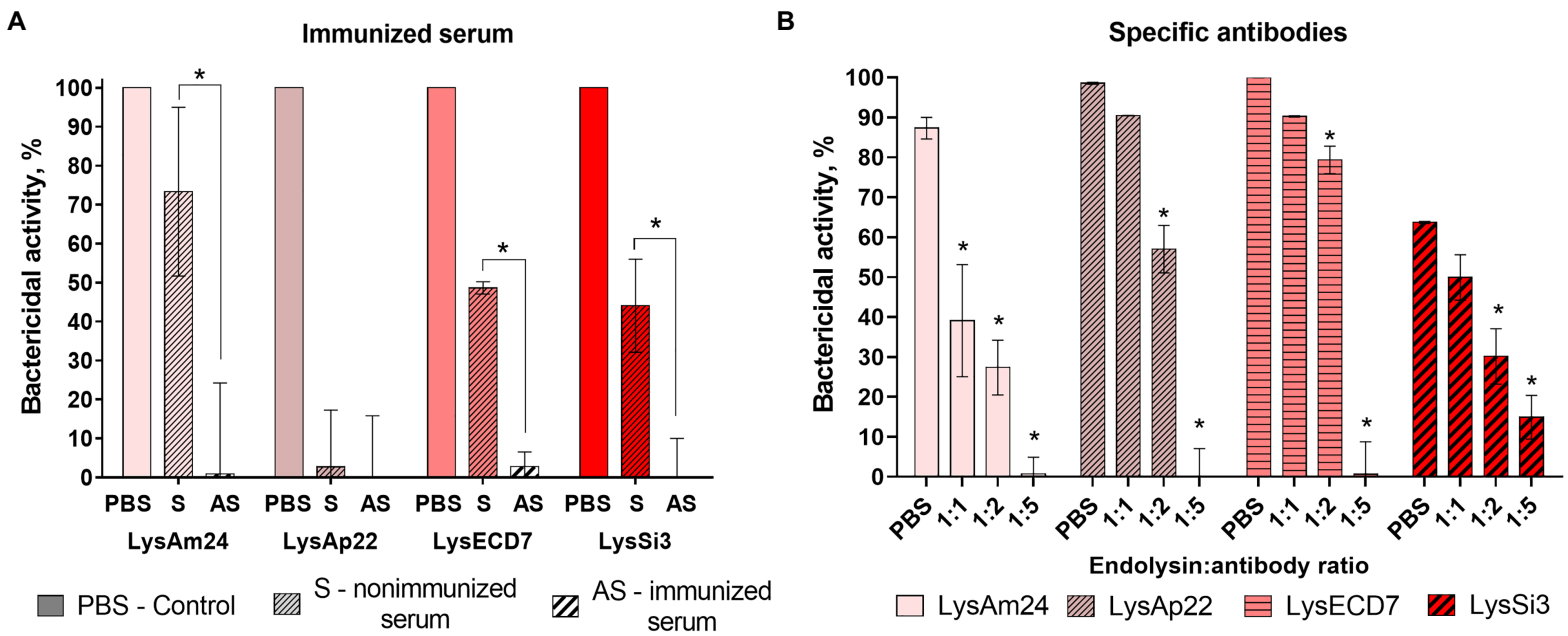
The neutralization effects of immunized serum **(A)** and specific antibodies **(B)** toward four endolysins. LysAm24, LysECD7, LysAp22, and LysSi3 were preincubated with endolysin-immunized rabbit serum, nonimmunized serum, PBS solution, or affinity-purified antibodies for 10min at 37°C. Each mixture was then added to the growing culture of *A. baumannii* Ts 50-16, and CFU count was estimated after 30-min incubation. Data are shown as mean±SD (one-way ANOVA). Asterisk (^*^) indicates significant difference in activity compared to nonimmunized serum **(A)** or control in PBS **(B**; *p* < 0.05).

The neutralization effect specific to LysAm24, LysAp22, LysECD7, and LysSi3 antibodies on their antibacterial activity was tested in the presence of hyperimmune serum and purified antibodies (Figure 3).

The presence of hyperimmune rabbit serum completely inhibited the antibacterial activity of all enzymes. However, the activity of 100μg/ml of endolysins in nonimmunized serum was significantly lower than in PBS (73, 47, and 44% for LysAm24, LysECD7, and LysSi3, correspondingly) and LysAp22 did not kill bacterial cells at all. Apparently, this is due to the inhibitory effect of the serum itself.

Therefore, the neutralization effect of purified antibodies was investigated to eliminate the serum effect. The addition of specific antibodies in mass ratios of 1:1 and 1:2 to reaction significantly decreased the antibacterial effect of LysAm24, LysAp22, LysECD7, and LysSi3. Moreover, a 1:5 mass ratio that corresponds approximately to the 1:1 molar ratio completely inhibits endolysin activity.

### Impact of Endolysins on the Normal Flora of the Gastrointestinal Tract

#### Interaction of Endolysins With Human Gut Microbiota *in vitro*

**Figure 4:**
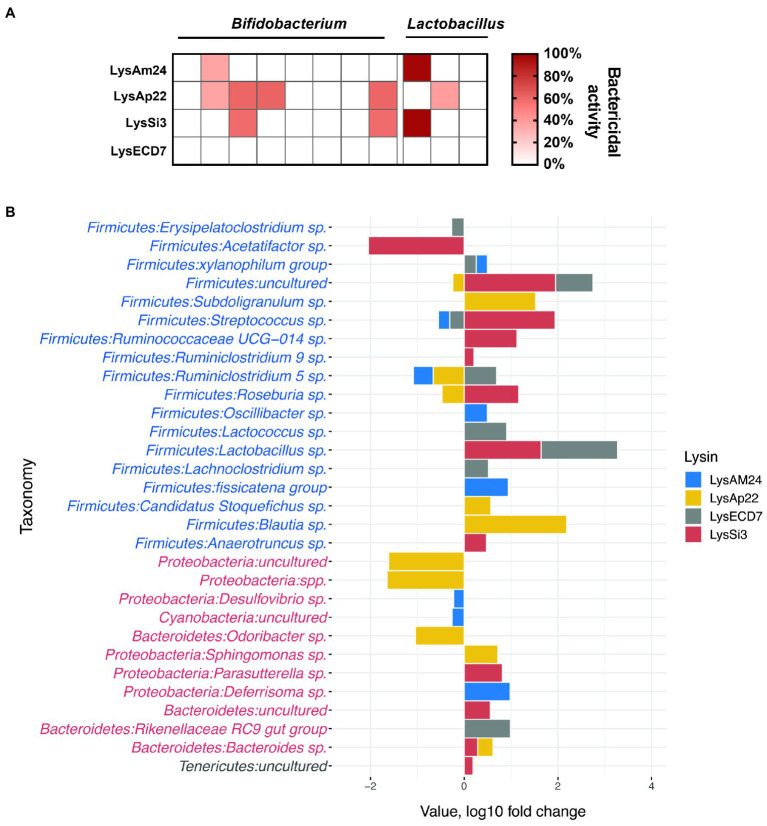
Impact of endolysins on the normal bacterial flora. **(A)** *In vitro* activity of LysAm24, LysECD7, LysAp22, and LysSi3 against *Bifidobacterium* spp. and *Lactobacillus* spp. strains. Activity spectra were assessed at 100-μg/ml endolysin concentration after 30-min incubation with bacterial suspension in a liquid medium. **(B)** Intestine microflora changes in mice under endolysin administration compared to control buffer (Tris–HCl) injections on the seventh day of experiment. Blue and red taxa indicate Gram-positive and Gram-negative bacteria correspondingly.

The *in vitro* impact on microbiota representatives was tested on strains of Gram-positive *Bifidobacterium* spp. and *Lactobacillus* spp. (Supplementary Table S1). Several strains were susceptible to endolysin LysAm24, LysAp22, and LysSi3 exposure (Figure 4A). LysAp22 was the most active against investigated strains and influenced the growth of five from 11 microbiota representatives. However, LysECD7 did not influence bacterial counts. The *Lactobacillus* genus was more susceptible to endolysin treatment *in vitro* and reached 98–99% of bacterial growth elimination for LysAm24 and LysSi3.

#### Interaction of Endolysins With Gut Microbiota in the Murine Model

Endolysins’ impact on the normal flora was also assessed in a murine model. Endolysin solutions in the concentration of 500μg/mouse were administered intraperitoneally every day for 5days. The composition of the parietal microbiome was estimated on the seventh day of experiment (2days after the last injection), and sequencing of intestinal fragments with feces was carried out, which showed changes in the microbiome from Tris–HCl buffer injections, illustrated in Figure 4B. In general, the identified bacteria of the gastrointestinal tract corresponded to those previously described in the literature as a healthy core gut microbiota (Wang et al., 2019). The main phyla that underwent quantitative changes under the endolysins administration were Proteobacteria and Bacteroidetes (Gram-negative) as well as Firmicutes (Gram-positive; Figure 4B). When comparing microflora profiles, mostly the increased growth of bacteria was observed, among which Lachnospiraceae, Lactobacillaceae, and Ruminococcaceae families were prevalent. While the most pronounced bacterial profile changes (both increase and decrease in 16S relative abundance) were shown under the administration of LysSi3, we detected a significant bacterial decrease for LysSi3 (Lachnospiraceae: *Acetatifactor*) and LysAp22 (Porphyromonadaceae, Caulobacteraceae, and Methylocystaceae families; Supplementary Figure S4). There was no decrease in *Bifidobacterium* genus *in vivo*, in contrast to *in vitro* data, while *Lactobacillus* tended to increase after the use of LysSi3 and LysECD7. Interestingly, no significant differences were found in opportunistic Gram-negative pathogens like *Escherichia* or *Klebsiella*. Thus, the experimental data confirm moderate endolysins’ effect on microbiome when administered parenterally.

### *In vivo* Efficacy of Endolysins

#### Skin Wound Model

Skin infection of outbred mice was induced with subcutaneous injection of *K. pneumoniae* Ts 141-14 suspension. The open wound was formed after the abscess rupture within 24h PI. Then infected areas were either left untreated, treated with control buffer (20mM Tris–HCl, pH 7.5), or treated with solutions of each investigated endolysin in the concentration of 500μg/mouse. The mice were treated epicutaneously once a day for 5days.

**Figure 5:**
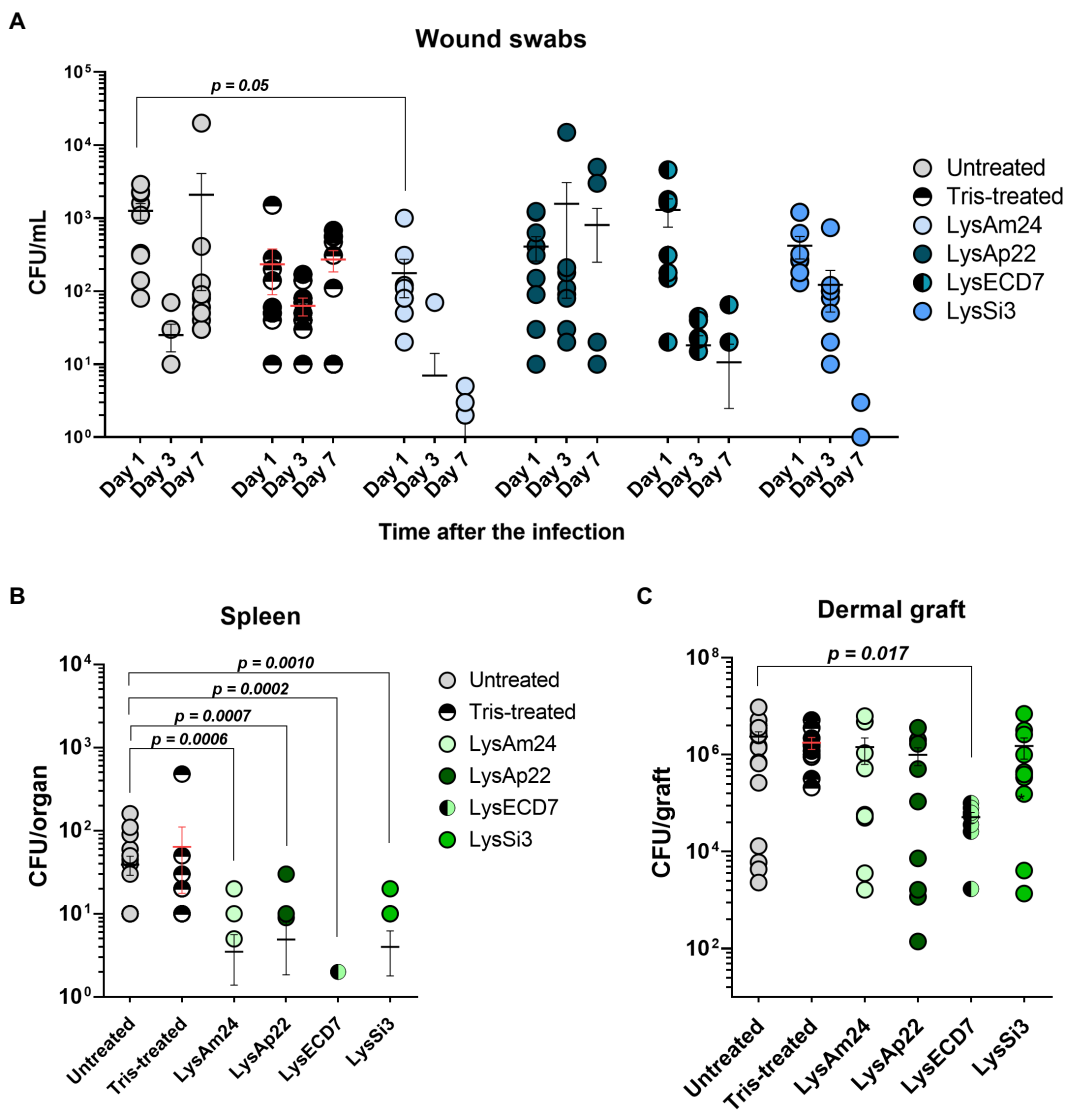
Skin wound model of outbred mice infected with the *K. pneumoniae* Ts 141-14 strain and epicutaneously treated with control buffer or 500-μg/ml endolysins solutions. **(A)** Bacterial counts in the wounds resulting from swabs of the infected areas. Significant differences are shown as *p* values; otherwise, no statistical difference was found (two-way ANOVA with Sidak multiple-comparison test). Spleen **(B)** and dermal graft **(C)** microbial load on the seventh day of the experiment. Significant differences are shown as *p* values; otherwise, no statistical difference was found (Mann–Whitney test). The resulting CFU/mL, CFU/graft, or CFU/organ values from each animal are plotted as individual points. Data are shown as mean±SEM.

The wound sizes and their microbial contamination were assessed in dynamics as well as spleen and dermal graft bacterial load (Figure 5).

The mean wound sizes in untreated animals were 3.8, 5.0, and 3.8mm in diameter on 1, 3, and 7days, respectively (data not shown). Tris-treated animals showed 3.4-, 4.0-, and 3.5-mm wound sizes on 1, 3, and 7days, respectively. Therefore, during the experiment, the size of the wounds in the control groups did not change significantly and no spontaneous wound healing was observed. At the same time, it was shown that administration of endolysin solutions results in a significant reduction of the wound size or ever-complete wound healing from the third day after the beginning of treatment. This reduction was 3.5 and 3.2mm for LysAm24; 3.0 and 2.3mm for LysECD7; 2.4 and 3.2mm for LysSi3; and 2.7 and 3.2mm for LysAp22 on 3 and 7days, respectively, compared to untreated animals on the same day. So, the pronounced wound healing was observed in all experimental groups and it was 60.5% for LysECD7 and 84.2% for LysAm24, LysSi3, and LysAp22 on the seventh day of the experiment.

The bacterial count after inoculation of wound swabs is shown in Figure 5A. Wound contamination in untreated mice on 1, 3, and 7days after infection was 1.2×10^3^, 2.5×10^1^, and 2.1×10^3^CFU/ml, respectively, which can be explained by nonsignificant infection healing and its further regression. Tris-treated mice showed a stable infectious process, and bacterial counts were 2.3×10^2^, 6.3×10^1^, and 2.7×10^2^CFU/ml on 1, 3, and 7days, respectively. Endolysin administration resulted in different antibacterial effects. LysAp22 did not show any significant microbial reduction in swabs, although wound sizes in this group were significantly lower. LysAm24, LysECD7, and LysSi3 showed pronounced bacterial reduction dynamics; in all three groups, more than 50% of animals had sterile wound swabs at the last time point (60% for LysAm24, 50% for LysECD7, and 70% for LysSi3), having mean values of, in CFU/ml, 1.1, 12.1, and 0.4 for LysAm24, LysECD7 and LysSi3, respectively. In total, microbial contamination reduction was 2.2–3.7 log_10_ compared to the untreated group on the seventh day.

On the seventh day of the experiment, the mouse dermal grafts and spleens were also investigated for microbial contamination. It was shown that local wound infection was generalized as bacteria were found in animals’ spleens (Figure 5B). Control groups showed a mean of 40 and 64CFU/organ for untreated and buffer-treated mice. All endolysins groups had significantly reduced bacterial counts or absence of bacteria in the spleen. Treatment with LysAm24 reduced the CFU/organ to 3.5, LysECD7 to 0.3, LysSi3 to 4.0, and LysAp22 to 4.9. Thus, endolysins prevented infection systematization reducing the bacterial load in the spleen for 0.9–2.14 log_10_ CFU compared to untreated animals.

Dermal graft homogenates contained 2.4×10^6^CFU/graft in the untreated group and 1.8×10^6^CFU/graft in buffer-treated mice (Figure 5C). LysAm24, LysSi3, and LysAp22 administration did not significantly reduce contamination, and CFU/graft was 1.4×10^6^, 1.5×10^6^, and 9.9×10^5^, respectively. Nevertheless, LysECD7 reduced bacterial count to 5.1×10^4^CFU/graft, which is a 1.7 log_10_ CFU reduction compared to untreated mice.

#### Burn Wound Model

Burn wound infection of Wistar rats was performed with a heated copper plate and infected with *P. aeruginosa* Ts 38-16 suspension. At 24h, PI burns were either left untreated or treated with control buffer (20mM Tris–HCl, pH 7.5) or treated with solutions of each investigated endolysin in the concentration of 2,500μg/animal. The rats were treated epicutaneously once a day for 5days.

**Figure 6:**
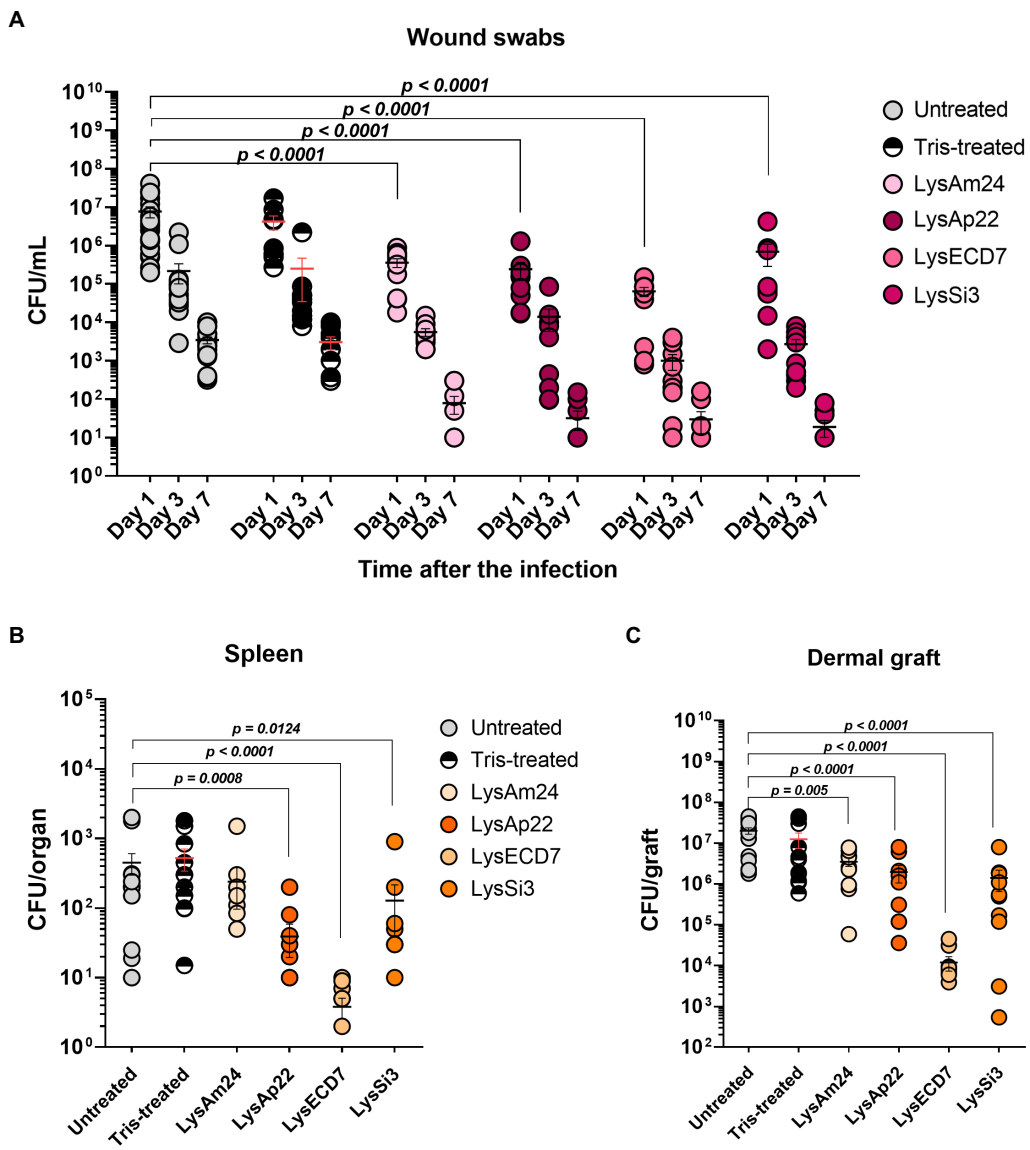
Burn wound model of Wistar rats infected with the *P. aeruginosa* Ts 38-16 strain and epicutaneously treated with control buffer or 2,500-μg/ml endolysin solutions. **(A)** Count of culturable bacteria in the wound swabs of the infected areas. Significant differences are shown as *p* values; otherwise, no statistical difference was found (two-way ANOVA with the Sidak multiple-comparison test). Spleen **(B)** and dermal graft **(C)** microbial contamination (CFU/organ) on the seventh day of the experiment are shown. Significant differences are shown as *p* values; otherwise, no statistical difference was found (Mann–Whitney test). The resulting CFU/mL, CFU/graft, or CFU/organ values from each animal are plotted as individual points. Data are shown as mean±SEM.

Microbial contamination of infected areas of untreated animals was 7.7×10^6^, 2.2×10^5^, and 3.5×10^3^, respectively, on 1, 3, and 7days after infection (Figure 6A). Tris-treated rats showed 4.2×10^6^, 2.5×10^5^, and 3.1×10^3^CFU/ml bacterial load on 1, 3, and 7days, respectively. The infectious process was stable in control groups; self-healing was not observed. Treatment with endolysin solutions resulted in positive dynamics of bacterial count reduction in all experimental groups, especially on the seventh day, when more than 40% of animals had a sterile burn surface (40% for LysAm24, 50% for LysECD7, LysSi3, and LysAp22) with mean values of 79, 32, 19, and 30CFU/ml for LysAm24, LysECD7, LysSi3, and LysAp22, respectively, which is a 1.6–2.3 log_10_ reduction.

Dermal grafts and spleen investigations were performed on the seventh day of the experiment. Spleens of infected animals were contaminated with *P. aeruginosa* so burn infection was generalized. Control groups showed means of 4.5×10^2^ and 5.2×10^2^CFU/organ for untreated and buffer-treated rats, respectively (Figure 6B). All endolysin groups, except LysAm24-treated animals, had up to 100-fold reduced bacterial count or absence of bacteria in the spleen and, therefore, prevented systematization of an infectious process. Treatment with LysECD7 reduced CFU/organ to a mean value of 3.8, LysSi3 to 128, and LysAp22 to 39.

Dermal grafts in control groups contained 2.0×10^7^CFU/graft in untreated animals and 1.3×10^7^CFU/graft in Tris-treated animals (Figure 6C). All endolysins significantly reduced contamination; the CFUs/graft were 3.5×10^6^, 1.4×10^6^, 1.2×10^4^, and 2.0×10^6^ for LysAm24, LysECD7, LysSi3, and LysAp22, respectively. Therefore, LysECD7 reduced bacterial count most pronounced, a 3.2 log_10_ reduction compared to approximately 1.0 log_10_ reduction in case of other endolysins.

## Discussion

Numerous endolysin studies confirm the *in vitro* effectiveness of these enzymes and describe valuable advantages of their use to combat multiple Gram-positive or -negative bacterial pathogens. However, very few of the studies are devoted to *in vivo* application and just several molecules are in clinical trials (Abdelrahman et al., 2021; Grabowski et al., 2021; Schmelcher and Loessner, 2021). Such a trend does not promote the acceleration of the development and implication of endolysin-based drugs into clinical practice. Moreover, it remains unclear what is the potential of using these molecules, especially for the treatment of drug-resistant Gram-negative bacteria-induced infections, which are the limitations and advantages of endolysin administration as individual antimicrobial therapy or in combination with antibiotics.

We provide a comprehensive study of four different unmodified Gram-negative bacteria-targeting endolysins, LysAm24, LysAp22, LysECD7, and LysSi3, devoted to their *in vitro* and *in vivo* potentials. The antibacterial activity kinetics showed that three out of four investigated molecules (LysAm24, LysAp22, and LysECD7) have similar dynamics of bacterial count reduction, although LysAp22 is more vigorous acting within the first 10min of incubation. LysSi3 acted significantly more slowly, although finally all proteins killed 100% of bacterial cells. These results are in consistency with previously published data, where up to 10^8^CFU/ml of the Gram-negative bacterial species were completely eliminated within 5min–1h by different endolysins (Raz et al., 2019; Wang et al., 2020; Chen et al., 2021). Since endolysins are proteins and can be prone to proteolytic degradation within the organism, it is highly desirable that they can remain in an active state at the injection site for a time sufficient for the implementation of antibacterial activity. Pharmacokinetics of unmodified Gram-positive hosting endolysin SAL200 shows the effective half-life *t_1/2_* within 15–25min after a single intravenous infusion of 1–10mg/kg dose (Jun et al., 2017). The time-killing curves of LysAm24, LysAp22, and LysECD7 show that although the complete elimination of bacteria occurs after an hour, the activity begins to manifest itself within the first 5–10min. Dependent on the lysin concentration, this may be sufficient for a local bactericidal effect before the significant degradation of the enzyme.

Although topical treatment strategies are much more common for endolysins and other lytic enzymes, systemic use also seems to be possible and effective enough (Lood et al., 2015; Raz et al., 2019; Wu et al., 2019). The broad spectrum of the bactericidal activity of endolysins under the study against both planktonic cells and bacterial films supports the possibility of their use to treat deep soft tissue lesions and abscesses, as well as polymicrobial infections of implanted devices or wound surfaces, associated with bacterial film formation (Lebeaux et al., 2014; Vestby et al., 2020). Antibiofilm activity is widespread among the Gram-negative acting endolysins. Thus, the disruption of *A. baumannii* (Chen et al., 2021), *P. aeruginosa* (Guo et al., 2017), or other bacterial species (Yuan et al., 2021) biofilms in a concentration-dependent manner was previously shown. Our results indicated that significantly greater concentrations of enzymes are needed for pronounced antibiofilm activity, compared to planktonic cells, but the observed effectiveness of LysAm24, LysAp22, LysECD7, and LysSi3 was enough to eliminate the BF to the threshold level, regardless of the biofilm-forming species. Microscopic investigation of biofilms grown on glass slides and exposed to endolysins showed bacterial film integrity disruption and significant bacterial cell rupture although some non-disrupted cells were also observed. It was shown that in our case these bacteria were capable of growing on an agar medium, with exception of LysAm24, so complete disruption of biofilm integrity does not necessarily mean the bacteria’s death. While the mechanisms of biofilm disruption by Gram-negative endolysins are not fully understood, it is unlikely that the effects are limited by the peptidoglycan interactions and the degradation could be associated with nonspecific interactions with matrix components.

During the safety experiments, we detected neither cytotoxic effect of endolysins on eukaryotic cells and red blood cells nor resistance development in bacteria toward LysAm24, LysAp22, LysECD7, and LysSi3. Earlier, no endolysin cytotoxicity against lung epithelial cells (Kim et al., 2020; Chen et al., 2021) or inhibitory effects on osteocyte-like cells were detected (Kuiper et al., 2021). Together with the previously published data (Briers et al., 2014; Zhang et al., 2016), this indicates that their application does not raise significant safety concerns, but should be approved in preclinical and clinical trials. At the same time, it is worth noting that we have shown the possibility of neutralizing antibody formation after animals’ immunization, which was capable of significantly reducing the antibacterial activity of enzymes. All endolysin-specific antibodies purified out of hyperimmune sera completely inhibited *in vitro* bactericidal activity in a 1:5 mass ratio or 1:1 molar ratio. This finding is opposite to data existing for Gram-positive bacteria-targeting endolysins whose activity in hyperimmune serum did not change (Rashel et al., 2007; Zhang et al., 2016; Yang et al., 2017). It is suggested that due to the presence of the cell wall-binding domain (CBD) in the structure, Gram-positive bacteria-targeting endolysins bind to neutralizing antibodies with less affinity compared to bacterial cell wall binding (Murray et al., 2021). On the opposite, Gram-negative bacteria-targeting endolysins often contain a single catalytic domain without CBD and, therefore, cannot counteract with bacterial cell walls, masking it from antibody neutralization. Thus, the reversible lysin–antibody interactions predominate. These results pose some potential obstacles to systemic endolysin-based drug development especially for administration into the bloodstream with long repeated courses. It remains unclear how much of the antibodies of hyperimmune rabbit sera obtained with the use of an adjuvant can be comparable in their neutralizing qualities with antibodies formed with the repeated use of finished dosage forms containing endolysin. Nevertheless, our results show that the neutralizing effects should be studied in detail in the development of antibacterials based on endolysins at the stage of preclinical and clinical studies.

Another aspect of safety concern is the effect of endolysin-based drugs on nontarget microorganisms, including the normal microbiota of patients (Becattini et al., 2016). In the case of Gram-positive targeting endolysins, this is avoided due to the high specificity of the action of such enzymes, which is frequently limited by one species or even the strain of the microorganism (Murray et al., 2021). Broad spectra of action of Gram-negative-targeting endolysins suggest that they can cause dysbiotic complications affecting the commensal microbial consortium. Since the intestinal flora is most susceptible, representatives of normal microflora that are part of probiotic drugs intended for correction of microflora were used to study the effect of endolysins. LysAm24, LysAp22, and LysSi3 significantly affected the growth of several strains, especially *Lactobacillus* representatives. At the same time, LysECD7 did not show any antibacterial effect against microflora *in vitro*. In general, the activity of endolysins against *Bifidobacterium* spp. and *Lactobacillus* spp. was significantly lower than against the Gram-negative genus. The 5-fold parenteral injections of investigated endolysins in *mice* have also resulted in bacterial changes. The increased growth of both Gram-negative and Gram-positive species was mainly observed, as indicated with 16S sequencing data. Only two of the investigated endolysins significantly reduced the abundance of commensal bacterial species compared to control group animals: LysSi3 reduced gram-positive Lachnospiraceae and LysAp22 inhibited bacteria from three Gram-negative families (Porphyromonadaceae, Caulobacteraceae, and Methylocystaceae). Although the moderate effects were mostly shown for all enzymes under investigation, these results indicate that no general uniform rules are applicable for different endolysins, and during the preliminary studies of preparations, it is important to assess the spectra of their action both against the target microorganisms and against the most common commensal species.

The effectiveness of Gram-negative infection treatment with endolysins was confirmed in two animal models. It was shown that administration of multiple epicutaneous endolysins on the wound and burn infections results in different degrees of antibacterial effect, but, in all cases, we observed a decreased microbial contamination, increased wound healing rates, and prevention of generalization of the infection. Different approaches for endolysin-based therapy applications were screened using the animal models. For example, the efficacy toward *P. aeruginosa* local lung and skin infection resulted in a more than 2-log_10_ CFU decrease by treatment with 100–300μg of PlyPa91 endolysin (Raz et al., 2019), and LysAB2-KWK endolysin was effective against *A. baumannii* systemic infection of *Galleria mellonella* larva in a 5-μg dose (Chen et al., 2021). More extensive data could be found for Gram-positive bacteria-caused local wound or burn infections, where it was shown that significantly lower doses (5–100μg of enzymes) are required to achieve the same or more pronounced therapeutic effect (Yang et al., 2017; Cheng et al., 2018; Wang et al., 2020).

Although these results are encouraging, now it is obvious that the effectiveness of unmodified Gram-negative bacteria-targeting enzybiotics is not enough to use them as self-therapy antibacterials. In the case of native, unmodified enzymes, their combination with antibiotics seems to be much more promising. For example, the synergistic effects of endolysins and antibiotics (colistin) *in vitro* and *in vivo* therapies were shown (Schirmeier et al., 2018; Abdelkader et al., 2020; Blasco et al., 2020). If developers succeed in defining an appropriate dosing schedule (beyond a single dose) as a basis for successful clinical studies for patients with confirmed infection due to drug-resistant pathogens or who experience recurrent or relapse infections, endolysins may provide an adjunctive therapy option. Another solution lies in the scope of endolysin bioengineering modifications and optimization to obtain specified properties or improve operational characteristics (Antonova et al., 2020; Gutiérrez and Briers, 2021).

To sum up, we have shown that LysAm24, LysAp22, LysECD7, and LysSi3 are capable of quickly eliminating Gram-negative bacteria *in vitro*, possess a wide spectrum of action, and are able to destroy the biofilms and significantly reduce contamination of the wound and burn skin surfaces, limiting the generalization of local infections. In terms of safety, these enzymes do not contribute to the development of short-term resistance, are not cytotoxic, and do not significantly affect the normal intestinal microflora *in vivo*. These properties provide a perspective basis for the development of preparations for the treatment of local infections, as well as for short-term use in systemic infections.

Endolysin-based drug development is an ambitious area of research, especially on account of raising bacterial resistance. However, insufficient information about their effectiveness in various animal models and clinical effectiveness complicates a decision-making process concerning the applicability of this alternative approach in antibacterial therapy. Studies of four different lysins show that, although they differ structurally, there are general patterns of their efficacy and safety, which means that the conclusions presented in this article can be applied and extended to some level to other lytic enzymes under investigation. An individual antibacterial therapy, especially systemic application, can be challenging due to their high molecular weight and proteinaceous nature, formation of neutralizing antibodies, rapid elimination, and pharmacokinetics. However, different studies and our data indicate that this class of antibacterial agents can increase the effectiveness of antibiotics, decrease therapeutic doses, and reduce the development and spread of antibiotic resistance.

## Data Availability Statement

The datasets presented in this study can be found in online repositories. The names of the repository/repositories and accession number(s) can be found at: https://www.ncbi.nlm.nih.gov/genbank/, APD20282.1, CCH57765.1, ASJ80195.1, and ARK07361.1.

## Ethics Statement

The animal study was reviewed and approved by the Ethics Committee of the State Research Center for Applied Microbiology and Biotechnology, Obolensk, Russia.

## Author Contributions

DV, NA, and VG conceived of the study and wrote, reviewed, and edited the manuscript. DV, NA, VP, AA, and IG provided the methodology. NA, IG, VY, AL, MN, EU, NS, MF, AV, and AL contributed to the investigation process. NA and AP provided formal analysis. VZ, TR, SY, and AT contributed to the funding acquisition and provided resources. VG and VM supervised the work. DV, NA, and AP wrote the original draft of the manuscript. All authors contributed to the article and approved the submitted version.

## Funding

This research study was supported by the Ministry of Health of the Russian Federation carried out in the frame of State Contract no. 0373100122119000013 of 15 May 2019 with the Center for Strategic Planning of the Ministry of Health of the Russian Federation.

## Conflict of Interest

NA, DV, AT, and VG are the authors but not the patent holders of the following patents issued according to the results of this work (RU): RU 2730613 C1 “Antibacterial composition (embodiments) and use of protein as antimicrobial agent directed against bacteria *Pseudomonas aeruginosa*, *Klebsiella pneumoniae*, *Escherichia coli*, *Salmonella typhi*, and *Staphylococcus haemolyticus* (embodiments)”; RU 2730614 C1 “Antibacterial composition (embodiments) and use of protein as antimicrobial agent directed against bacteria *P. aeruginosa*, *K. pneumoniae*, *Escherichia coli*, *Salmonella typhi*, and *Staphylococcus haemolyticus* (embodiments)”; RU 2730615 C1 “Antibacterial composition (embodiments) and use of protein as antimicrobial agent directed against Gram-negative bacteria: *P. aeruginosa*, *Acinetobacter baumannii*, *K. pneumoniae*, and *Salmonella typhi* (embodiments)”; and RU 2730616 C1 “Antibacterial composition (embodiments) and use of protein as an antimicrobial agent directed against *A. baumannii* bacteria (embodiments).”

The remaining authors declare that the research was conducted in the absence of any commercial or financial relationships that could be construed as a potential conflict of interest.

## Publisher’s Note

All claims expressed in this article are solely those of the authors and do not necessarily represent those of their affiliated organizations, or those of the publisher, the editors and the reviewers. Any product that may be evaluated in this article, or claim that may be made by its manufacturer, is not guaranteed or endorsed by the publisher.
