# Supplementary material for: Discovering the Potentials of Four Phage Endolysins to Combat Gram-Negative Infections
Source: Front Microbiol. 2021 Oct 13;12:748718. doi: 10.3389/fmicb.2021.748718 (PMC8548769; doi:10.3389/fmicb.2021.748718)
Supplement: Supplementary file 1 [file Data_Sheet_1.DOCX]

Supplementary Material


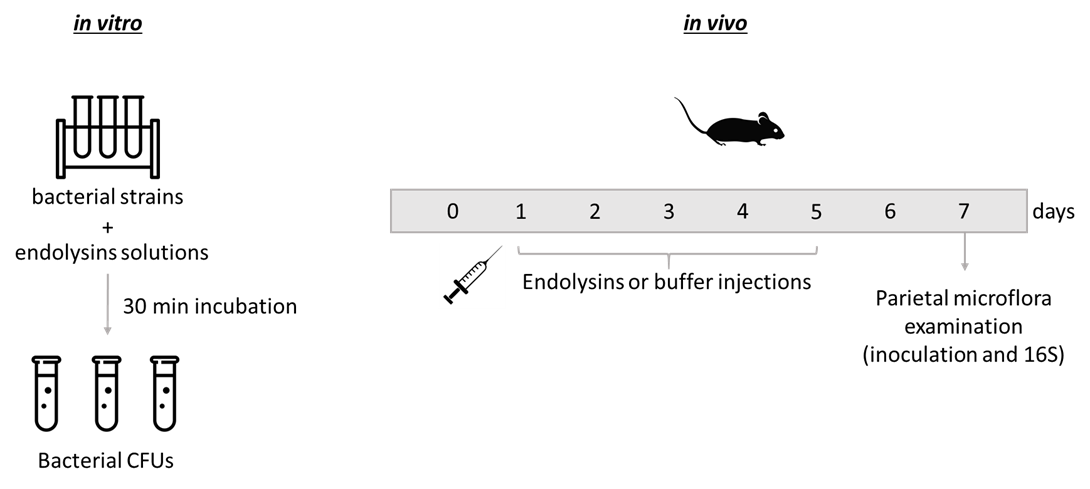


**Supplementary Figure 1.** Experimental design of endolysins intestinal microbiome impact *in vitro* and *in vivo* assessment.


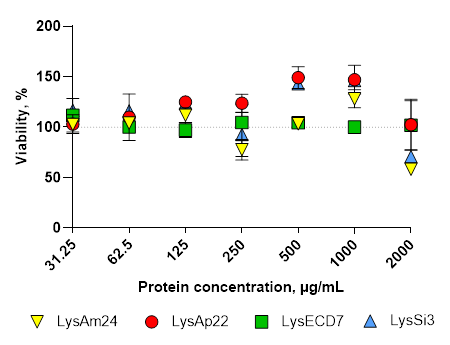


**Supplementary Figure 2.** Endolysins cytotoxicity assay. The effect of LysAm24, LysAp22, LysECD7 and LysSi3 in different concentrations on the viability of HEK293 cells was determined with an MTT assay after 1 h exposure. The mean values with SD are shown, all experiments were performed in triplicate.


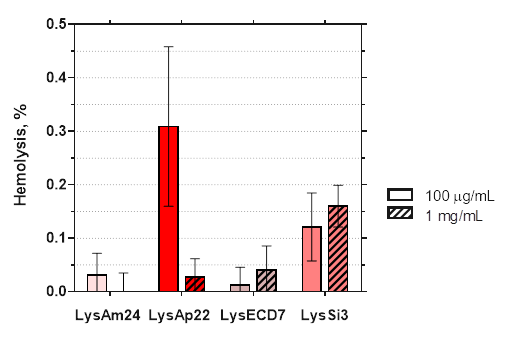


**Supplementary Figure 3.** Hemolytic activity of LysAm24, LysAp22, LysECD7 and LysSi3 against human red blood cells after 1 h exposure. The mean values with SD are shown, all experiments were performed in triplicate.


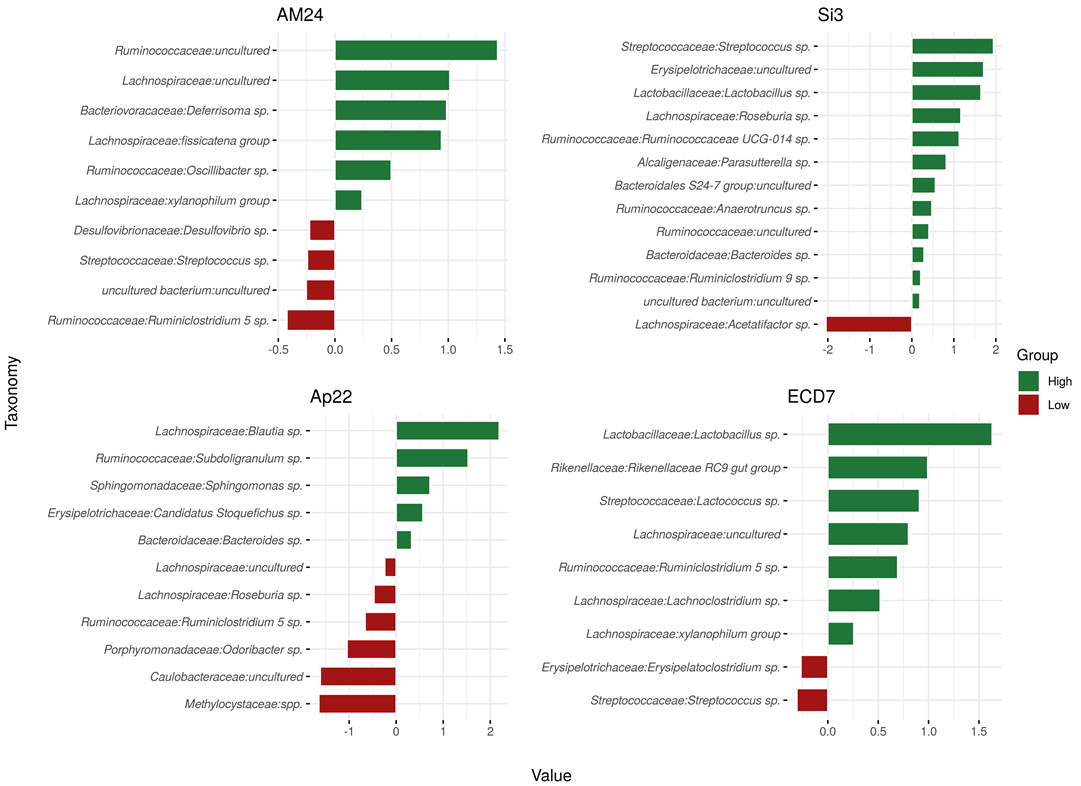


**Supplementary Figure 4.** Parietal microflora changes in mice. 16S rRNA gene sequencing of intestine fragments samples collected on the 7^th^ day of experiment (2 days after last injection of LysAm24, LysECD7, LysAp22 and LysSi3 in 0.5 mg/mouse).

**Supplementary Table 1.** Bacterial strains used in the study. R – resistant, MR – moderately resistant, n/d – no data.

| **Strain** | **Drug resistance** | **Source** | **Method of identification** | **Assay** |
| --- | --- | --- | --- | --- |
| *Klebsiella pneumoniae* 1 | R: AMP, SAM, CXM, CAZ, CRO, CPZ/SUL, FEP, IPM, AMK, GEN, TOB, CIP, TET, CHL, SXT | Hospital strain, inpatient hospital | Cultural, morphological and biochemical properties | Determination of spectrum of activity |
| *Klebsiella pneumoniae* 2 | R: AMP, SAM, CXM, CAZ, CRO, CPZ/SUL, FEP, GEN, TOB, CIP, TET, CHL, SXT;  MR: ETP, IPM, TGC |  |  |  |
| *Klebsiella pneumoniae* 3 | R: AMP, SAM, CXM, CAZ, CRO, CPZ/SUL, FEP, ETP, IPM, GEN, TOB, CIP, TET, CHL, SXT;  MR: TGC |  |  |  |
| *Klebsiella pneumoniae* 4 | R: AMP, SAM, CXM, CAZ, CRO, CPZ/SUL, FEP, ETP, IPM, GEN, TOB, CIP, TET, TGC, CHL, SXT |  |  |  |
| *Klebsiella pneumoniae* 5 | R: AMP, SAM, CXM, CAZ, CRO, FEP, GEN, TOB, CIP, TET, CHL, SXT |  |  |  |
| *Klebsiella pneumoniae* 6 | R: AMP, SAM, CXM, CAZ, CRO, CPZ/SUL, FEP, ETP, IPM, GEN, TOB, CIP, TET, CHL, SXT;  MR: TGC |  |  |  |
| *Klebsiella pneumoniae* 7 | R: AMP, SAM, CXM, CAZ, CRO, CPZ/SUL, FEP, IPM, GEN, TOB, CIP, CHL, SXT;  MR: ETP |  |  |  |
| *Klebsiella pneumoniae* 8 | R: AMP, SAM, CXM, CAZ, CRO, CPZ/SUL, FEP, IPM, GEN, TOB, CIP, CHL, SXT;  MR: ETP |  |  |  |
| *Klebsiella pneumoniae* 9 | R: AMP, SAM, CXM, CAZ, CRO, CPZ/SUL, FEP, ETP, IPM, GEN, TOB, CIP, TET, TGC, CHL, SXT;  MR: AMK |  |  |  |
| *Klebsiella pneumoniae* 10 | R: AMP, SAM, CXM, CAZ, CRO, CPZ/SUL, FEP, ETP, IPM, GEN, TOB, CIP, TET, CHL |  |  |  |
| *Klebsiella pneumoniae* 11 | R: AMP, SAM, CXM, CAZ, CRO, CPZ/SUL, FEP, ETP, IPM, GEN, TOB, CIP, TET, TGC, CHL |  |  |  |
| *Klebsiella pneumoniae* 12 | R: AMP, SAM, CXM, CAZ, CRO, CPZ/SUL, FEP, GEN, TOB, CIP, TET, CHL, SXT;  MR: ETP, IPM, TGC |  |  |  |
| *Klebsiella pneumoniae* 13 | R: AMP, SAM, CXM, CAZ, CRO, CPZ/SUL, FEP, ETP, IPM, AMK, GEN, TOB, CIP, SXT;  MR: CHL |  |  |  |
| *Klebsiella pneumoniae* 14 | R: AMP, AMC, CTX, CAZ, CPZ/SUL, FEP, IPM, MEM, AMK, CIP |  |  |  |
| *Klebsiella pneumoniae* 15 | R: AMP, SAM, CXM, CAZ, CRO, CPZ/SUL, FEP, ETP, IPM, AMK, GEN, TOB, CIP, CHL, SXT;  MR: TGC |  |  |  |
| *Klebsiella pneumoniae* 16 | R: AMP, SAM, CXM, CAZ, CRO, CPZ/SUL, FEP, ETP, IPM, GEN, TOB, CIP, CHL, SXT |  |  |  |
| *Klebsiella pneumoniae* 17 | Extended spectrum beta lactamases production;  R: AMP, AMC, CTX, CAZ, CPZ/SUL, FEP, IPM, GEN, CIP, SXT  MR: MEM |  |  |  |
| *Klebsiella pneumoniae* 18 | R: AMP, SAM, CXM, CAZ, CRO, CPZ/SUL, FEP, ETP, IPM, AMK, GEN, TOB, CIP, CHL |  |  |  |
| *Klebsiella pneumoniae* 19 | R: AMP, SAM, CXM, CAZ, CRO, CPZ/SUL, FEP, ETP, IPM, AMK, GEN, TOB, CIP, TET, TGC, CHL, SXT |  |  |  |
| *Klebsiella pneumoniae* 20 | n/d |  |  |  |
| *Klebsiella pneumoniae* Ts 104-14 | Extended spectrum beta lactamases production;  R: ETP, IPM, MEM | Hospital strain, outpatient hospital | Cultural, morphological and biochemical properties, MALDI-TOF | Biofilm reduction assay, determination of bacterial resistance |
| *Klebsiella pneumoniae* Ts 141-14 | R: AMP, CTX, CAZ,  MEM, GEN, CIP, TET, CHL | Hospital strain, inpatient hospital |  | Skin wound model |
| *Salmonella enterica*subsp.*enterica*serovar Enteritidis «Kaluga» | n/d | Animal infectious disease, chicken | Cultural, morphological and biochemical properties, serological typing | Determination of spectrum of activity |
| *Salmonella enterica*subsp.*enterica*serovar Infantis 4632 | n/d |  |  |  |
| *Salmonella enterica*subsp.*enterica*serovar Tiphimurium «Lo» | n/d | Animal infectious disease, pigeons |  |  |
| *Salmonella enterica*subsp.*enterica*serovar Enteritidis 3B | n/d | Animal infectious disease, chicken |  |  |
| *Salmonella enterica*subsp.*enterica*serovar Infantis 2511 | R: AMP, TET, CAZ, CLI |  |  |  |
| *Salmonella enterica*subsp.*enterica*serovar Tiphimurium 415 | R: CLI |  |  |  |
| *Salmonella enterica*subsp.*enterica*serovar Tiphimurium 1281 | n/d |  |  |  |
| *Salmonella enterica*subsp.*enterica*serovar Tiphimurium Ufa | n/d | Animal infectious disease, ducks |  |  |
| *Salmonella enterica*subsp.*enterica*serovar Tiphimurium 3 | n/d |  |  |  |
| *Salmonella enterica*subsp.*enterica*serovar Tiphimurium 24 | R: CRO | Animal infectious disease, chicken |  |  |
| *Salmonella enterica*subsp.*enterica*serovar Enteritidis 25 | R: SXT |  |  |  |
| *Salmonella enterica*subsp.*enterica*serovar Infantis 4631 | R: CHL, AMP, AMC, TET, CAZ, CLI |  |  |  |
| *Salmonella enterica*subsp.*enterica*serovar Infantis 4522 | R: AMC, AMP, CLI |  |  |  |
| *Salmonella enterica*subsp.*enterica*serovar Enteritidis «4B» | n/d |  |  |  |
| *Salmonella enterica*subsp.*enterica*serovar Infantis «Kuzn» | n/d |  |  |  |
| *Salmonella enterica*subsp.*enterica*serovar Dublin immobilin 2 | n/d | Animal infectious disease, calf |  |  |
| *Salmonella enterica*subsp.*enterica*serovar London N2 | n/d | Animal infectious disease, turkey |  |  |
| *Salmonella enterica*subsp.*enterica*serovar Tiphimurium G1 | n/d | Livestock wastes | Cultural, morphological and biochemical properties, MALDI-TOF |  |
| *Salmonella enterica*subsp.*enterica*serovar Infantis 1271 | n/d |  |  |  |
| *Salmonella enterica*subsp.*enterica*serovar Enteritidis SEМ4 | n/d |  | Cultural, morphological and biochemical properties |  |
| *Pseudomonas aeruginosa* 1 | R: CTX, CAZ, CPZ/SUL, FEP, MEM, AMK, GEN, CIP | Hospital strain, inpatient hospital | Cultural, morphological and biochemical properties, MALDI-TOF | Determination of spectrum of activity |
| *Pseudomonas aeruginosa* 2 | R: CTX, CAZ, CPZ/SUL, FEP, MEM, AMK, GEN, CIP |  |  |  |
| *Pseudomonas aeruginosa* 3 | R: CTX, CAZ, CPZ/SUL, FEP, MEM, AMK, GEN, CIP |  |  |  |
| *Pseudomonas aeruginosa* 4 | R: CTX, CAZ, CPZ/SUL, FEP, MEM, AMK, GEN, CIP |  |  |  |
| *Pseudomonas aeruginosa* 5 | R: CTX, CAZ, CPZ/SUL, FEP, MEM, AMK, GEN, CIP |  |  |  |
| *Pseudomonas aeruginosa* 6 | R: CTX, CAZ, CPZ/SUL, FEP, MEM, AMK, GEN, CIP |  |  |  |
| *Pseudomonas aeruginosa* 7 | R: CTX, CAZ, CPZ/SUL, FEP, MEM, AMK, GEN, CIP |  |  |  |
| *Pseudomonas aeruginosa* 8 | R: CTX, CAZ, CPZ/SUL, FEP, MEM, AMK, GEN, CIP |  |  |  |
| *Pseudomonas aeruginosa* 9 | n/d |  |  |  |
| *Pseudomonas aeruginosa* 10 | n/d |  |  |  |
| *Pseudomonas aeruginosa* 11 | n/d |  |  |  |
| *Pseudomonas aeruginosa* 12 | R: AMK, FEP, CAZ, C/T, CIP, GEN, IPM, LVX, MEM, TZP |  |  |  |
| *Pseudomonas aeruginosa* 13 | R: FEP, CAZ, C/T, CIP, GEN, IPM, LVX, MEM, TZP, TOB |  |  |  |
| *Pseudomonas aeruginosa* 14 | R: AMK, FEP, CAZ, CIP, GEN, IPM, LVX, MEM, TZP, TOB |  |  |  |
| *Pseudomonas aeruginosa* 15 | R: AMK, FEP, CAZ, C/T, CIP, GEN, IPM, LVX, MEM, TZP, TOB |  |  |  |
| *Pseudomonas aeruginosa* 16 | R: CAZ, CIP, GEN, LVX, MEM, TZP, TOB;  MR: AMK |  |  |  |
| *Pseudomonas aeruginosa* 1805 | R: FEP, CAZ, GEN, IPM, LVX, MEM, TZP, TOB |  |  |  |
| *Pseudomonas aeruginosa* 3086 | R: FEP, CAZ, C/T, CIP, GEN, IPM, LVX, MEM, TZP | Reference laboratory strain |  |  |
| *Pseudomonas aeruginosa* PА01 | n/d |  |  |  |
| *Pseudomonas aeruginosa* B-1304 | n/d |  |  |  |
| *Pseudomonas aeruginosa* Ts 38-16 | R: CPZ/SUL,  MR: MEM, AMK, CRO, CST | Hospital strain,  intensive care unit | Cultural, morphological and biochemical properties, MALDI-TOF | Biofilm reduction assay, burn wound model |
| *Escherichia coli* 96 | R: FEP, CAZ, CIP, LVX, AMP, CRO, CXM, SXT | Hospital strain, inpatient hospital | Cultural, morphological and biochemical properties, MALDI-TOF | Determination of spectrum of activity |
| *Escherichia coli* 532 | R: AMK, FEP, CAZ, CIP, GEN, IPM, LVX, MEM, TZP, AMP, CRO, CXM, SXT |  |  |  |
| *Escherichia coli* 502 | R: AMK, FEP, CAZ, C/T, CIP, GEN, IPM, LVX, MEM, TZP, AMP, CRO, CXM, ETP, TGC, SXT |  |  |  |
| *Escherichia coli* 125 | R: FEP, CAZ, CIP, LVX, MEM, TZP, AMC, AMP, SAM, CRO, CXM, SXT |  |  |  |
| *Escherichia coli* 636 | R: FEP, CAZ, C/T, CIP, LVX, AMC, AMP, SAM, CRO, CXM, SXT |  |  |  |
| *Escherichia coli* 16 | R: AMK, FEP, CAZ, C/T, CIP, LVX, AMP, CRO, CXM, SXT |  |  |  |
| *Escherichia coli* 110 | R: FEP, CAZ, CIP, GEN, IPM, LVX, MEM, TZP, AMP, CRO, CXM, ETP, TGC, SXT |  |  |  |
| *Escherichia coli* 158 | R: AMK, FEP, CAZ, C/T, CIP, GEN, IPM, LVX, MEM, TZP, AMC, AMP, SAM, CRO, CXM, ETP, TGC, SXT |  |  |  |
| *Escherichia coli* 452 | R: FEP, CAZ, CIP, LVX, AMP, CRO, CXM, SXT |  |  |  |
| *Escherichia coli* 510 | R: AMK, FEP, CAZ, C/T, CIP, LVX, AMP, CRO, CXM, ETP, TGC, SXT |  |  |  |
| *Escherichia coli* 663 | R: FEP, CAZ, CIP, GEN, IPM, LVX, MEM, TZP, AMP, CRO, CXM, ETP, TGC, SXT |  |  |  |
| *Escherichia coli* 185 | R: FEP, CAZ, CIP, GEN, IPM, LVX, MEM, TZP, AMC, AMP, SAM, CRO, CXM, ETP, TGC, SXT |  |  |  |
| *Escherichia coli* 515 | R: AMK, FEP, CAZ, C/T, CIP, LVX, AMP, CRO, CXM, SXT |  |  |  |
| *Escherichia coli* 682 | R: FEP, CAZ, CIP, LVX, AMC, AMP, SAM, CRO, CXM, SXT |  |  |  |
| *Escherichia coli* 108 | R: FEP, CAZ, CIP, GEN, IPM, LVX, MEM, TZP, AMC, AMP, SAM, CRO, CXM, ETP, TGC, SXT |  |  |  |
| *Escherichia coli* 503 | R: FEP, CAZ, CIP, LVX, AMP, CRO, CXM, SXT |  |  |  |
| *Escherichia coli* 632 | R: AMK, FEP, CAZ, C/T, CIP, LVX, AMP, CRO, CXM, ETP, TGC, SXT |  |  |  |
| *Escherichia coli* 179 | R: FEP, CAZ, CIP, GEN, IPM, LVX, MEM, TZP, AMC, AMP, SAM, CRO, CXM, ETP, TGC, SXT |  |  |  |
| *Escherichia coli* 201 | R: AMK, FEP, CAZ, C/T, CIP, LVX, AMC, AMP, SAM, CRO, CXM, ETP, TGC, SXT |  |  |  |
| *Escherichia coli* 533 | R: FEP, CAZ, CIP, LVX, AMP, CRO, CXM, SXT |  |  |  |
| *Acinetobacter baumannii* 145 | R: SAM, CAZ, CRO, CPZ/SUL, FEP, IPM, CIP;  MR: GEN | Hospital strain, inpatient hospital | Cultural, morphological and biochemical properties, MALDI-TOF |  |
| *Acinetobacter baumannii* 402 | R: SAM, CAZ, CRO, CPZ/SUL, FEP, IPM, GEN CIP, TET, TGC |  |  |  |
| *Acinetobacter baumannii* МА 65 | R: FEP, GEN, IPM, SAM, CRO;  MR: CAZ |  |  |  |
| *Acinetobacter baumannii* 474 | R: CTX, CAZ, CPZ/SUL, FEP, IPM, GEN CIP, SXT |  |  |  |
| *Acinetobacter baumannii* 869 | R: FEP, CAZ, GEN, IPM, TOB, SAM, CRO, TGC |  |  |  |
| *Acinetobacter baumannii* Bor | R: CAZ, GEN, IPM, TOB, SAM, CRO, TGC, SXT;  MR: FEP |  |  |  |
| *Acinetobacter baumannii* РА | R: FEP, CAZ, GEN, IPM, TOB, SAM, CRO, TGC, SXT |  |  |  |
| *Acinetobacter baumannii* 67GKB | R: FEP, CAZ, GEN, IPM, SAM, CRO, TGC |  |  |  |
| *Acinetobacter baumannii* B-05 | R: FEP, CAZ, GEN, IPM, SAM, CRO, TGC, SXT |  |  |  |
| *Acinetobacter baumannii* Gar | R: CAZ, GEN, IPM, TOB, SAM, CRO;  MR: FEP | Hospital strain, intensive care unit | Cultural, morphological and biochemical properties |  |
| *Acinetobacter baumannii* Ts 50-16 | n/d | Hospital strain,  intensive care unit | Cultural, morphological and biochemical properties | Antibacterial activity assay, biofilm reduction assay, microscopy, determination of bacterial resistance, evaluation of neutralizing antibodies |
| *Enterobacter* sp*.* 1 | n/d | Hospital strain, outpatient hospital | Cultural, morphological and biochemical properties, MALDI-TOF | Determination of spectrum of activity |
| *Enterobacter* sp*.* 2 | n/d |  |  |  |
| *Enterobacter* sp*.* 3 | n/d |  |  |  |
| *Enterobacter* sp*.* 4 | n/d |  |  |  |
| *Enterobacter* sp*.* 5 | n/d |  |  |  |
| *Enterobacter* sp*.* 6 | n/d |  |  |  |
| *Enterobacter* sp*.* 7 | n/d |  |  |  |
| *Enterobacter* sp*.* 8 | n/d |  |  |  |
| *Enterobacter* sp*.* 9 | n/d |  |  |  |
| *Enterobacter* sp*.* 10 | n/d |  |  |  |
| *Bifidobacterium bifidum* OV-19 | n/d | Healthy human microbiota representative | Cultural, morphological and biochemical properties | *In vitro* microbiota impact assay |
| *Bifidobacterium bifidum* 791 | n/d |  |  |  |
| *Bifidobacterium longum* OV-20 | n/d |  |  |  |
| *Bifidobacterium longum* Ya-3 | n/d |  |  |  |
| *Bifidobacterium longum* B379M | n/d |  |  |  |
| *Bifidobacterium breve* OV-12 | n/d |  |  |  |
| *Bifidobacterium adolescentis* GО-13 | n/d |  |  |  |
| *Bifidobacterium infantis* 73-15 | n/d |  |  |  |
| *Lactobacillus helveticus* NK-1 | n/d |  |  |  |
| *Lactobacillus helveticus/casei* К3sch24 | n/d |  |  |  |
| *Lactobacillus casei* КНМ-12 | n/d | Dairy product |  |  |

Antibacterial agents abbreviations: Amikacin – AMK; Amoxicillin/Clavulanic Acid – AMC; Ampicillin – AMP; Ampicillin/Sulbactam – SAM; Cefepime – FEP; Cefoperazone/Sulbactam – CPZ/SUL; Cefotaxime – CTX; Ceftazidime – CAZ; Ceftolozane/Tazobactam – C/T; Ceftriaxone – CRO; Cefuroxime – CXM; Chloramphenicol – CHL; Ciprofloxacin – CIP; clindamycin – CLI; Colistin – CST; Ertapenem – ETP; Gentamicin – GEN; Imipenem – IPM; Levofloxacin – LVX; Meropenem – MEM; Piperacillin/Tazobactam – TZP; Tetracycline – TET; Tigecycline – TGC; Tobramycin – TOB; Trimethoprim/Sulphamethoxazole – SXT.

Antibiotic susceptibility analysis of bacterial strains was performed by the broth microdilution method using Mueller-Hinton broth, according to ISO recommendations (ISO 20776-1. Clinical laboratory testing and in vitro diagnostic test systems—Susceptibility testing of infectious agents and evaluation of performance of antimicrobial susceptibility testing devices—part 1. Geneva, Switzerland: International Organization for Standardization, 2006). Results were interpreted according to The European Committee on Antimicrobial Susceptibility Testing. Breakpoint tables for interpretation of MICs and zone diameters. Version 11.0, 2021.
